# Physics-Informed Neural Networks Meet Multimodal Large Language Models: Biomechanical Simulation in Aortic Aneurysm

**DOI:** 10.34133/cbsystems.0658

**Published:** 2026-09-02

**Authors:** Shichao Zhu, Mieradilijiang Abudupataer, Yulin Wang, Hongqiang Zhang, Hao Lai, Kai Zhu, Yongxin Sun, Nan Chen

**Affiliations:** Department of Cardiac Surgery and Shanghai Institute of Cardiovascular Diseases, Zhongshan Hospital, Fudan University, Shanghai 200032, P.R. China.

## Abstract

Axial dissections of the thoracic artery are common causes of death in people diagnosed with aortic dissection; however, decisions to intervene on ascending thoracic aortic patients are determined by the size of the ascending thoracic aorta based on its diameter. Diameter-based criteria fail to take into consideration the biomechanical properties of the aorta as well as other characteristics of the patient, and finite element analysis (in determining aortic wall stresses) would ideally address the issues associated with today’s diameter-based criteria. A novel computational solution, termed BioPINN-LM, integrates 2 computational methods for the rapid real-time prediction of aortic wall stress: a physics-informed neural network (PINN) trained with mechanical simulation data to predict wall stress and a multimodal large language model that uses output data from the PINN along with image-based geometry descriptors to provide interpretable risk assessments from both ends of the aorta. Comparison of the PINN to the reference database of finite element method results demonstrates that the PINN produces an average wall stress prediction error of 8.34 kPa (6.12% relative error) for a blood pressure of 120/80 mmHg, with an average prediction time of 0.83 s per geometry compared to 38.6 min for the traditional finite element method. The conversational component of BioPINN-LM achieves 87.4% agreement with board-certified specialist recommendations on a benchmark of 200 simulated clinical scenario vignettes. As a simulation-based proof of concept, these results suggest that integrating mechanistic simulations with language-based inquiry may complement biomechanical decision-making for ascending thoracic aortic aneurysms; clinical validation on real patient cohorts with longitudinal outcomes is deferred to future work.

## Introduction

Ascending thoracic aortic aneurysm (ATAA) is the gradual enlargement of the thoracic aorta, which causes most people to not have symptoms; however, a small group of patients will suffer from either dissection or rupture, leading to fatality either before they arrive at the hospital (estimated at 40%) or during hospital admission (at approximately 25% even in high-volume centers [1,2]). The most significant risk criterion/indicator for ATAA is the maximum transverse diameter (approximately 5.5 cm for tricuspid valves and 5.0 cm for bicuspid valves) [3,4]; however, recent studies have stated that approximately 60% of type A dissections happen below the surgical intervention threshold [1,5], indicating a major flaw; while the maximum diameter indicates the larger scale of how large an aneurysm is, it does not reflect how the area around the aorta is experiencing different levels of stress and strain; therefore, this is what will eventually cause rupture.

The local rupture is caused when the local area around the aorta reaches a level of stress greater than the strength of the local tissue surrounding the aorta [6–8]. By using finite element analysis (FEA) in combination with anatomically specific patient images and a model to simulate local stresses, many studies have demonstrated that it is possible to obtain detailed information on the stresses within abdominal [9,10] and thoracic [11–14] aortic aneurysms. One of the major barriers to using FEA in routine clinical evaluation of patients when it comes to ATAA is that there is no standardized method for conducting each of the steps of FEA: manual data input, surface generation, volume mesh, defining material properties, and conducting FEA. The time associated with these steps is 4 to 8 h per case for a qualified specialist [15,16]. Fully automated methods such as FEAorta [17,18] still have the issue that the time taken to generate results is measured in minutes rather than seconds, making them less useful in practice because they do not allow for interactive exploration of hemodynamic states.

Physics-informed neural networks (PINNs) integrate governing partial differential equations (PDEs) into the loss function of the neural network, thereby removing the requirement for the traditional mesh-and-solve loop in computational fluid dynamics (CFD) methods [19,20]. PINNs have demonstrated their potential to produce accurate approximations of CFD solutions for vascular biomechanics through initial applications focused on velocity and pressure fields in well-defined, idealized abdominal aortic aneurysm (AAA) geometries [21,22]. For example, relative errors for CFD solutions obtained using PINNs were under 5%, and compute times were reduced by many orders of magnitude compared to those of traditional CFD methods. In the field of operator learning, deep operator networks (DeepONets) extend this concept further by mapping the boundary conditions of the problem to the complete solution field [23]. However, to date, no research has used PINNs to analyze the structural stress of ATAAs, where the hyperelastic and anisotropic properties of the structure, combined with the complex geometry of the ascending aorta, create unique challenges that are not present in laminar flow situations.

Recent advances in multimodal large language models (LLMs) are making great strides in the diagnosis and treatment of cardiac patients using LLMs to combine multiple modalities of medical information [24–26]. In cardiology, LLMs have shown promising results for interpreting electrocardiography images [27], echocardiographic viewpoints [28,29], and responding to questions about the management of patients with cardiovascular disease (CVD) based on the latest guidelines [30]. LLMs offer the ability to create comprehensive and useful explanations combining various forms of data, such as patient geometry, hemodynamics, comorbidity profiles, and guideline thresholds, into natural language that can be readily questioned through conversational interfaces [31,32]. Currently, no LLM has been developed to accept outputs from physics-based simulations to generate biomechanical recommendations. Consequently, when asked about the evolution and management of patients with AAA, even the most sophisticated medical LLMs will typically revert to diameter-based heuristics.

These 2 types of research address complementary obstacles in predicting stresses on the human body. While the PINN approach allows for real-time stress predictions without requiring a large amount of computation and the multimodal language model (LLM) approach allows for interpretation of simulation results by decision-makers, we require solutions to at least 3 formidable challenges in order to achieve a “bridge” between these 2 approaches. These challenges include (a) developing an appropriate PINN model for accurately simulating the quasi-incompressible, hyperelastic material properties of the aorta under normal and hypertensive conditions; (b) designing a representation of the PINN’s 3-dimensional (3D) stress field that is usable as a conditioning input by LLMs without incurring catastrophic information losses; and (c) developing instruction-tuning datasets that will allow for LLMs to learn how to evaluate and reason about mechanical risk factors rather than simply reproducing the guidelines for the allowed maximum diameter of an arterial segment.

This paper presents BioPINN-LM, a hybrid computational framework—intended as a research prototype and interactive biomechanical simulation assistant rather than a clinically actionable decision-support tool—that addresses these 3 difficulties. We highlight the following contributions:

• a PINN formulation for estimating wall stress in ATAA that incorporates the Holzapfel–Gasser–Ogden (HGO) hyperelastic material model and the governing equations of equilibrium into a single neural network architecture, resulting in a 6.12% mean relative error when comparing the results of our model with that of FEA on validation synthetic geometry given normotensive pressure (“PINN branch for aortic wall stress estimation” section)
• a systematic encoding of stress fields in the form of a MechToken that represents a 3D compression of von Mises stress and principal stress distributions into a fixed-length tensor suitable for use with the multimodal input interfaces of LLMs (see the “MechToken encoding” section)
• a multimodal LLM module that has been trained using instructions, which has the capability to condition jointly on the imaging-derived geometric features of ATAA and the generated MechTokens and therefore will provide conversational style risk assessment based on standard guidelines with 87.4% agreement to the recommendations of clinical experts over 200 clinical cases (“Multimodal LLM branch for conversational decision support” section)

**Fig. 1.**
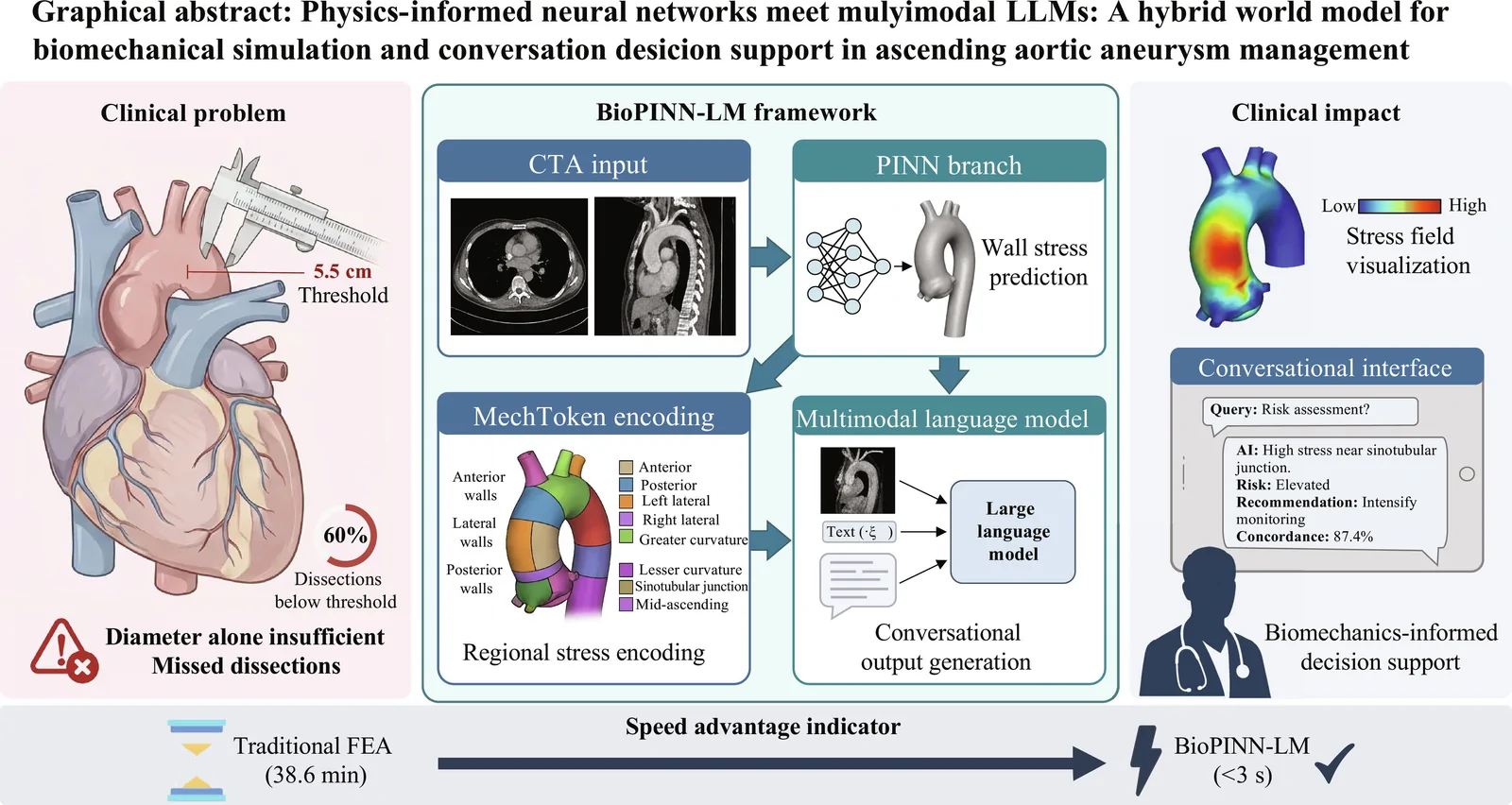
This graphical representation details the BioPINN-LM framework. To the left of the image: one of the clinical limitations in treating patients with ascending thoracic aortic aneurysms that only use diameter as a measure of risk; to the right of the image: examples of how this research prototype could, after future clinical validation, complement biomechanical reasoning through stress visualization and conversational interpretation. The speed advantage indicator shows how much faster (38.6 min vs. less than 3 s) the proposed biomechanical approach could provide information relative to what has previously been available through traditional finite element analysis (FEA).

Fig. 1 illustrates how computed tomography angiography (CTA)-derived geometry, PINN-predicted wall stress, and multimodal language modeling are integrated to provide biomechanically informed conversational decision support in less than 3 s.

This paper is divided into several sections. Related Work discusses prior art with respect to aortic biomechanical measurement through modeling, Materials and Methods explains how BioPINN-LM is built, Results contains results of computational experimentation using BioPINN-LM, and Discussion describes limitations and suggests areas for future research, while Conclusion summarizes all the findings and conclusions of the paper.

## Related Work

### Biomechanical modeling of aortic aneurysms

The analysis of the stress distribution of the aortic wall using finite element methods has undergone considerable advancement in the last 20 years. Raghavan and Vorp [33] originally presented a hyperelastic isotropic model of aortic wall tissue. This model is still used as a baseline by most companies developing aortic wall finite element models for use in AAA. Gasser et al. [34] presented an alternative model known as the HGO model, which can also account for the orientation of collagen fibers and provides an anisotropic representation of the aortic wall. The HGO model has become the standard model for the representation of thoracic aorta [7,35]. For patient-specific models of ATAAs, Wisneski et al. [11] demonstrated the utility of deriving patient-specific FEAs by obtaining zero-pressure geometry using micro-computed tomography images and conducting tensile testing for the 27 samples they examined. They found that for the samples they tested, wall stress at peak stress correlated poorly with the maximum vessel diameter (ρ=0.324, P=0.099). Similarly, Krishnan et al. [12] used FEA techniques with in vivo MRI-derived models of ATAAs and established that neglecting the zero-pressure correction of the material increases the wall stress by about 15% to 30%. Martin et al. [13] offered a probabilistic failure metric based on a geometrical model of the aorta trained on biaxial test data from 79 patients. Most of the studies described above share the limitation that FEAs require many hours to prepare; therefore, cohort studies have generally included fewer than 100 patients, limiting the statistical power available to support clinical use [16].

### Neural surrogates for vascular biomechanics

Various research teams have conducted studies on the application of neural networks as surrogates to speed up various calculations currently performed by FEA. Rengarajan et al. [36] created a shallow neural network using 53 different geometric indices to predict the maximum stress experienced by the wall of 148 AAAs. Their model produced an $R^{2}$ value of 0.89 for symptomatic AAAs. The MultiViewUNet group [37] shifted from the traditional method of calculating wall shear stress to treating it as an image-to-image task; therefore, they projected AAA surfaces onto a 2-dimensional view of the body and achieved a normalized mean absolute error (MAE) rate of 0.362% when using synthetic geometries as input to their model. The third group, Chung et al. [38], developed a random forest classifier for AAAs using morphometric and biomechanical features. This classifier was validated on a cohort of 311 patients with a corresponding area under the receiver operating characteristic curve (AUC) value of 0.86. Unfortunately, all of these data-based surrogate models [39–41] fail to take into account the underlying factors that govern the physical behavior of AAAs. This presents challenges when simulating hypertensive crises or other abnormalities associated with the AAA, especially in terms of being able to extrapolate results beyond the range of input data used to train those models.

By incorporating PDEs into the loss function, PINNs can generate predictions that are physically accurate even when there is little training data available [19]. Karniadakis et al. [20] reviewed the use of PINNs across all types of fluid and solid mechanics applications but also recognized that cardiovascular (CV) hemodynamics is ideally suited for analysis using PINNs. Cruz-González et al. [21] compared the performance of physics-informed deep operator networks (PI-DeepONets), DeepONets, and PINNs on a 3D model of an idealized AAA flow and concluded that PI-DeepONets provided the best balance between accuracy and generalization. Ur Rehman et al. [22] applied PINNs for fluid–structure interactions to analyze aortic aneurysms with the geometry of Marfan syndrome and demonstrated close correlation with results from CFD for calculating wall shear stress (within 12% error). However, although PINNs have successfully been used for various types of fluid–structure interaction problems, no PINN has attempted to address structural (solid mechanics) stress analysis for ATAAs. The use of the hyperelastic constitutive model in ATAAs presents extra nonlinear difficulty due to the large deformations.

### LLMs in CV medicine

Considerable evidence exists in the literature that LLMs can be used effectively for the prediction of CV risk, for interpreting clinical practice guidelines, and for triaging patients with suspected CVD. Ferreira Santos et al. [30,42] systematically reviewed 35 published studies applying LLMs to the area of CVD and concluded that general-purpose LLMs, such as GPT-4, performed reasonably well in answering questions based on clinical practice guidelines, although they did not incorporate patient outcome data into their responses. In addition, a *The Lancet Digital Health* perspective paper [31] stated that the true transformative impact of LLMs on CV care will be achieved through the creation of architecture based on multimodal models that synthesize and integrate disparate forms of patient data, such as electronic health records, imaging studies, and wearable devices. EchoClip [28] is an example of this type of model that incorporates a vision–language interface and has been developed for the analysis of echocardiographic images. This model was able to classify views and determine the ejection fraction in zero-shot conditions with competitive accuracy. Nolin-Lapalme et al. [32] provided a detailed summary of the practical application of LLMs within cardiology and outlined the need for fine-tuning of LLMs with respect to specific areas of CV care, as well as beginning to form a “chain of thought” methodology in order to reduce hallucinations generated by these models.

There are many areas of research within the bionic and hybrid-system community where deep learning has been leveraged for tasks related to human skeletal motion detection and prediction, for example, skeleton-based human motion prediction and recognition of when someone interacts with other people or objects [43,44], electroencephalography-based brain–computer interfaces using transformers [45], physiological signals to identify emotional states [46], modeling cultural dynamics between cultures based on reinforcement learning [47], and using medical imaging techniques (i.e., MRI) to assess body composition [48]. There are signs of a convergence between neural computations (computers) and biological systems, but to date, there has been no research that couples a mechanistic physics engine for biomechanical assessment with conversational artificial intelligence (AI); this is the niche that BioPINN-LM fills.

## Materials and Methods

**Fig. 2.**
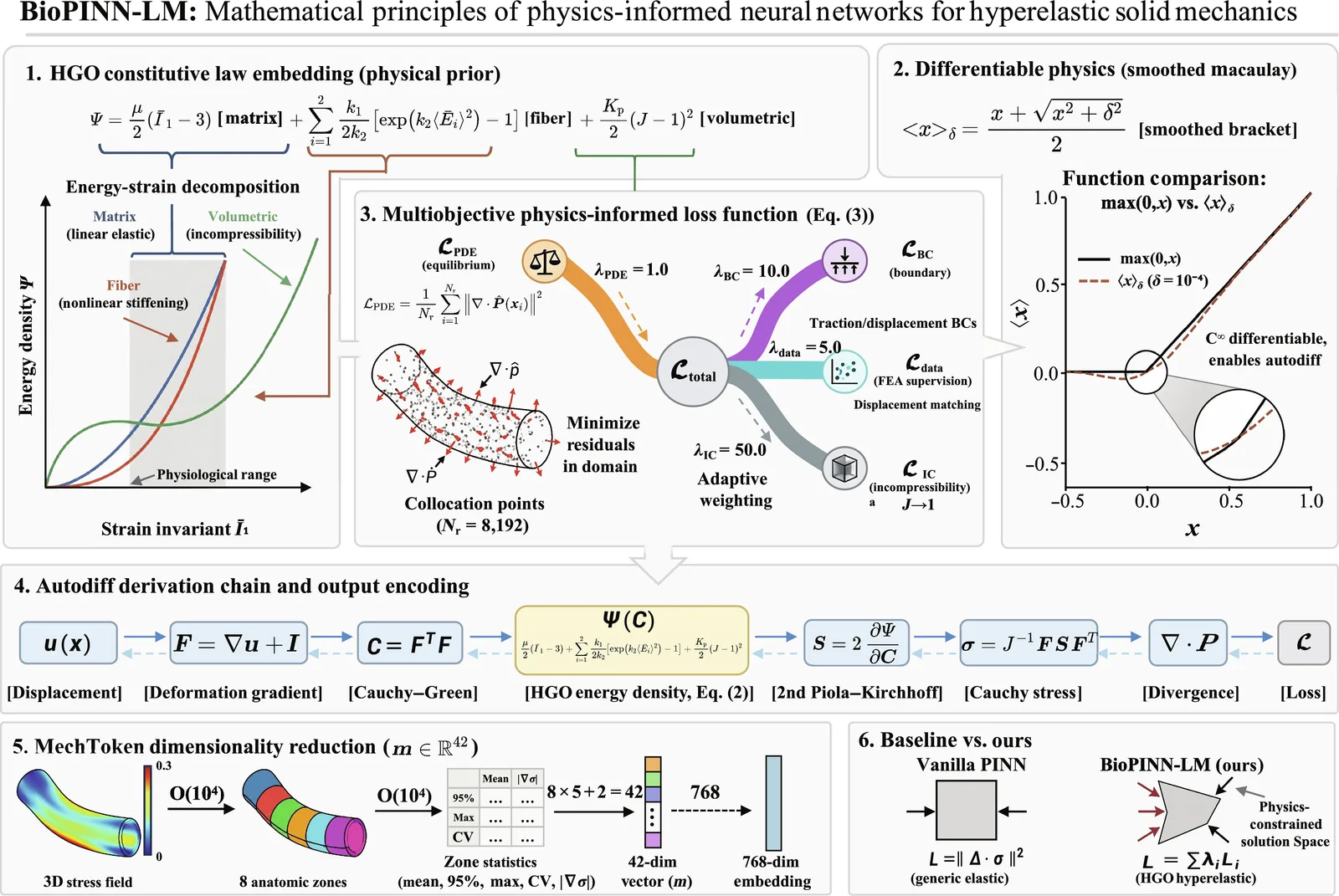
The BioPINN-LM physics-informed neural network (PINN) branch is established through a mathematical framework. Its components include (1) a 4-phase decomposition of Holzapfel–Gasser–Ogden (HGO) strain energy, (2) a smoothed Macaulay bracket for differentiable fiber activation, (3) a 4-term physics-informed loss with adaptive weighting, (4) an autodiff chain from displacement to Cauchy stress, (5) MechToken dimensionality reduction, and (6) a schematic comparison with the standard PINN.

Before covering the various elements, we provide an overall mathematical perspective of how the HGO property’s law, the smoothed Macaulay bracket, and the multiobjective loss function are combined to lead to Cauchy’s stress predictions. These stress predictions are then compressed into the 42-dimensional MechToken representation. Fig. 2 provides a schematic overview of this mathematical pipeline, tracing the path from HGO strain energy through the physics-informed loss to the final MechToken encoding.

### Problem formulation

An ATAA geometry Ω is recreated from CTA images. The aortic wall is treated as a hyperelastic material that has a pseudo-incompressible character, which is described by the equilibrium condition in the reference configuration (here, we have that b = body forces and can safely ignore them based on how the aortic wall is modeled):

$$\nabla \cdot P\left(x\right)+b\left(x\right)=0,\;x\in \Omega_{0}$$ (1)

Using the deformation gradient F=∇u+I, we identify the reference space $\Omega_{0}$ and the present space Ω. The current state of the vessel in space can be associated with the degree of displacement u that was applied to the reference structure ($\Omega_{0}$).

Based on the HGO model [34], the strain energy density can be expressed as

$$\Psi =\frac{\mu }{2}\left({\bar{I}}_{1}-3\right)+\sum_{i=1}^{2}\frac{k_{1}}{2k_{2}}\left[\text{exp}\left(k_{2}{\langle {\bar{E}}_{i}\rangle }^{2}\right)-1\right]+\frac{K_{p}}{2}{\left(J-1\right)}^{2}$$ (2)

where ${\bar{I}}_{1}=\mathrm{tr}\left(\bar{C}\right)$ is the first invariant of isochoric Cauchy–Green tensor $\bar{C}$, where μ is the shear modulus of the ground substance, and where $k_{1}$ and $k_{2}$ are the stiffness and nonlinearity parameters associated with the fibers, respectively. In addition, ${\bar{E}}_{i}=\kappa_{d}{\bar{I}}_{1}+\left(1-3\kappa_{d}\right){\bar{I}}_{4i}-1$; $J=\det\left(F\right)$ is the criterion imposed for near-incompressibility, via the volumetric penalty modulus $K_{p}$, which in this case has been set to 1.0 MPa (2 orders of magnitude larger than the shear modulus [15]); variations in $K_{p}$ (0.1 to 10 MPa) resulted only in a peak wall stress difference of less than 0.4%); as well as the new notation introduced through Macaulay brackets (x)=max(x,0).

The second Piola–Kirchhoff stress is given by S=2∂Ψ/∂C and can be expressed as the push-forward of the Cauchy stress: $\sigma =J^{-1}{\mathrm{FSF}}^{T}$.

Boundary conditions include an inner surface $\Gamma_{\text{in}}$ where the luminal pressure $p_{\mathrm{lum}}$ is applied as a follower load and at the fixed-to-the-surface $\Gamma_{\mathrm{fix}}$ boundaries of the aortic root and distal limits, where displacements are constrained. The goal of this study is to predict the entire Cauchy stress tensor, $\sigma \left(x\right)$, associated with arbitrary geometries of ATAA in conjunction with arbitrary pressure loading, followed by the summarization of the mechanical risk profile of the ATAA in an easily interpreted manner for language models.

**Fig. 3.**
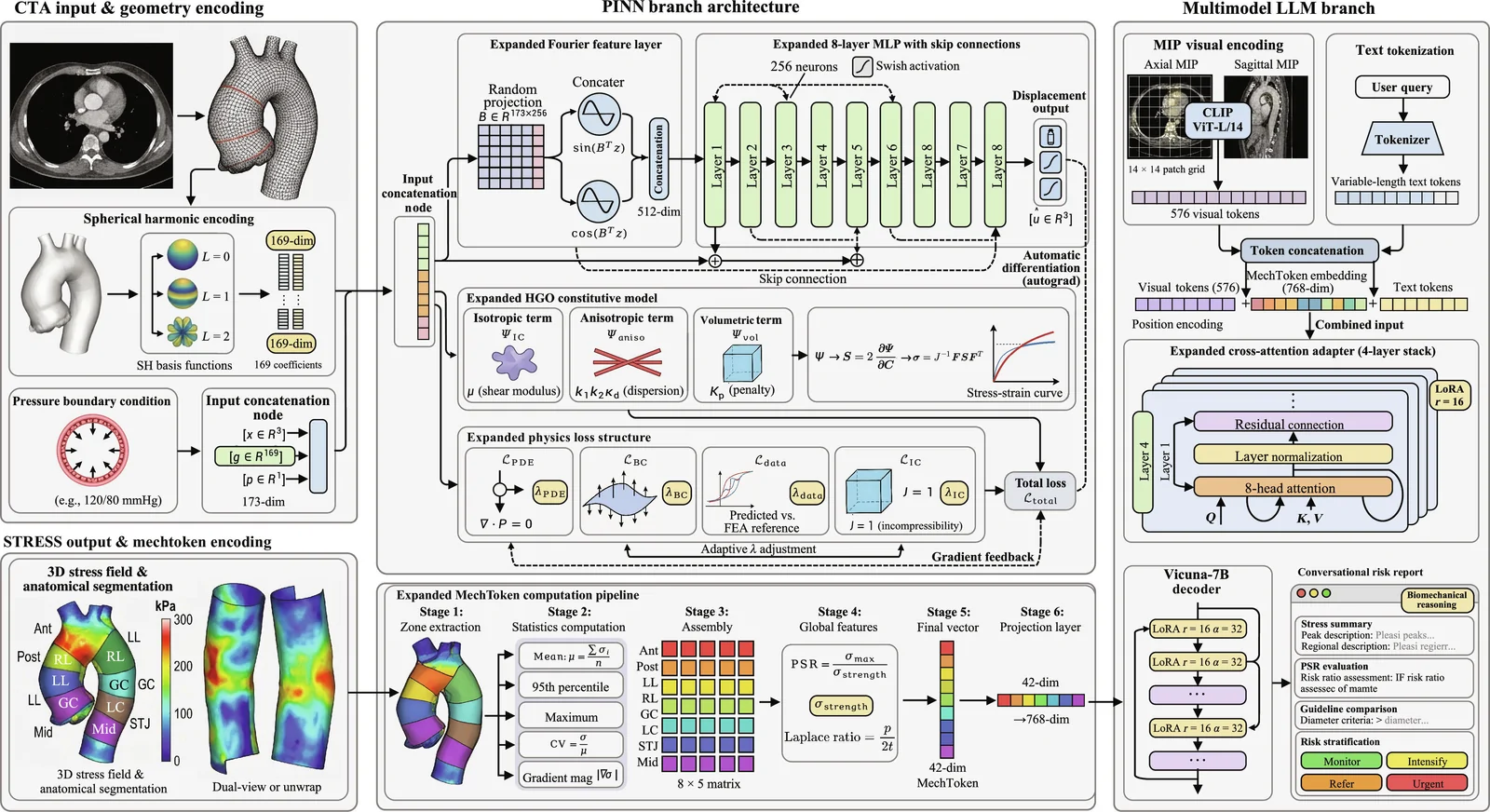
System architecture of BioPINN-LM. Left: computed tomography angiography (CTA)-derived ascending thoracic aortic aneurysm (ATAA) geometry encoded via spherical harmonics and concatenated with luminal pressure. Center: physics-informed neural network (PINN) branch with a Fourier feature layer, an 8-layer multilayer perceptron (MLP), and Holzapfel–Gasser–Ogden (HGO)-embedded loss producing a Cauchy stress field compressed into a 42-dimensional MechToken. Right: a multimodal large language model (LLM) branch fusing visual tokens, MechToken embeddings, and text tokens through a cross-attention adapter before the Vicuna-7B decoder generates a conversational risk report.

The architecture of BioPINN-LM is composed of 3 components. On the left, the CTA-derived ATAA luminal geometry is encoded via spherical harmonics and concatenated with the scalar luminal pressure to form the model input. The middle section of the diagram depicts the hidden layers of the neural network that generate a full Cauchy stress field, which is then compressed into a 42-dimensional vector called “MechToken”. The third and rightmost component of this architecture illustrates how the various modalities (e.g., visual tokens, MechToken embeddings, and text tokens) are fused together and then fed into a decoder (called Vicuna-7B) to generate a conversational risk report. Fig. 3 shows the overall design of this pipeline. This figure shows how the PINN-stress predictor, geometry encoding, MechToken compression, and LLM report generator work together as a single forward pass.

### PINN branch for aortic wall stress estimation

#### Network architecture

In the PINN branch, the 3D spatial coordinate x from ${\mathbb{R}}^{3}$, the geometry-conditioning vector g from ${\mathbb{R}}^{G}$, and the pressure scalar $p_{\mathrm{lum}}$ are combined as inputs to form a predicted displacement $\hat{u}\left(x;\;g,\;p_{\mathrm{lum}}\right)\in {\mathbb{R}}^{3}$. The values of g have been developed based on the shape of the ATAA geometry represented as a truncated spherical harmonic of the luminal surface of the geometry (for *L* = 12), yielding $G={\left(L+1\right)}^{2}=169$ coefficients available to represent a variety of complicated curvatures with a smooth differentiable representation.

The primary network is designed using a modification of the Fourier-based feature network [49] containing 8 hidden layers with sizes of 256 with activation functions chosen to be Swish. Layer 1 maps the input of $\left[x;\;g;\;p_{\mathrm{lum}}\right]$ into the Fourier feature domain through the random Fourier features with $\sigma_{f}=2.0$ and is intended to assist with convergence to the correct output location and minimize oscillatory output behavior produced from the asymmetric nature and high-frequency noise in the mechanical and fluid properties experienced at transitions from geometrically distinct shapes and features of the geometry (i.e., the location of the sinotubular junction [STJ] and the entire sinotubular segment). For layers 4 and 6, there are skip connections that allow the input into those layers to contain the original raw input values.

#### Physics-informed loss

The total loss is

$$L_{\text{total}}=\lambda_{\mathrm{PDE}}L_{\mathrm{PDE}}+\lambda_{\mathrm{BC}}L_{\mathrm{BC}}+\lambda_{\text{data}}L_{\text{data}}+\lambda_{\mathrm{IC}}L_{\mathrm{IC}}$$ (3)

where each term is defined below. Conceptually, the local mechanical equilibrium of the HGO-driven stress field is enforced by means of $L_{\mathrm{PDE}}$, while $L_{\mathrm{BC}}$ couples prediction with luminal pressure and proximal/distal kinematic constraints. Furthermore, $L_{\text{data}}$ anchors the solution to FEA reference displacements, when available. Finally, tissue near-incompressibility is enforced through the Jacobian penalty in accordance with $L_{\mathrm{IC}}$.

##### PDE residual loss

For each of the $N_{r}$ collocation points sampled within the volume of the wall,

$$L_{\mathrm{PDE}}=\frac{1}{N_{r}}\sum_{i=1}^{N_{r}}{\left\|\nabla \cdot \hat{P}\left(x_{i}\right)\right\|}^{2}$$ (4)

where $\hat{P}$ is produced from the predicted deformation through automatic differentiation of the HGO model (see Eq. (2)). In each training iteration using a large-volume, weight-sampling method, we select $N_{r}=8,192$ collocation points for each part geometry; in addition to this total number being approximately equal across all parts, the wall-thickness-weighted scheme results in a greater number of collocation points in areas with a low wall thickness (i.e., where stress gradients are the steepest and transitioning from wall to wall).

##### Smoothed Macaulay bracket

Although $\langle x\rangle$ is nondifferentiable (and therefore not amenable to automatic differentiation) at zero in the HGO model’s strain energy function (see Eq. (2)), we can approximate it smoothly with an infinitely differentiable function.

The following is the proposed approximation, where δ is a smoothing parameter:

$${\langle x\rangle }_{\delta }=\frac{x+\sqrt{x^{2}+\delta^{2}}}{2}$$ (5)

The approximation converges to the exact function as δ→0, is smooth, and therefore is differentiable everywhere. Selecting $\delta =10^{-4}$ ensures that the maximum deviation from the exact answer will be $\delta /2=5\times 10^{-5}$ and is small enough not to significantly affect our stress estimates ($O\left(10^{2}\right)$ kPa). This is demonstrated by the fact that varying δ between 10^−6^ and 10^−2^ yields less than 0.01% variation in the neural network’s stress predictions.

##### Boundary-condition loss

On the luminal surface, the traction boundary condition is enforced as

$$L_{\mathrm{BC}}=\frac{1}{N_{b}}\sum_{j=1}^{N_{b}}{\left\|\hat{P}\left(x_{j}\right){\hat{n}}_{j}+p_{\mathrm{lum}}JF^{-T}{\hat{n}}_{j}\right\|}^{2}$$ (6)

where ${\hat{n}}_{j}$ is the outward normal in the reference configuration. On $\Gamma_{\mathrm{fix}}$, a Dirichlet loss penalizes ${\left\|\hat{u}\right\|}^{2}$.

##### Data loss

When reference FEA solutions are available, a supervised data term is added:

$$L_{\text{data}}=\frac{1}{N_{d}}\sum_{k=1}^{N_{d}}{\left\|\hat{u}\left(x_{k}\right)-u_{k}^{\mathrm{FEA}}\right\|}^{2}$$ (7)

We use $N_{d}=2,048$ nodal displacement samples per available FEA solution.

##### Incompressibility constraint

To enforce near-incompressibility,

$$L_{\mathrm{IC}}=\frac{1}{N_{r}}\sum_{i=1}^{N_{r}}{\left(J\left(x_{i}\right)-1\right)}^{2}$$ (8)

We determined the loss weightings of $\lambda_{\mathrm{PDE}}=1.0$, $\lambda_{\mathrm{BC}}=10.0$, $\lambda_{\text{data}}=5.0$, and $\lambda_{\mathrm{IC}}=50.0$ using a grid search over 20 held-out test geometries. Also, we trained our models with Adam optimizer with an initial learning rate of 5 × 10^−4^ and cosine-annealing schedule for 200 epochs. We employed a step decay factor of 0.5 after every 40 epochs. In addition, we used the adaptive loss weighting strategy proposed by Wang et al. [50] whereby changes in loss weights deviated from those prescribed by Wang et al. every 10 epochs based upon the relative magnitudes of the gradients of each individual loss function (e.g., PDE, boundary-condition loss, data, and incompressibility-constraint loss).

#### Synthetic geometry generation

To generate a variety of training geometries, we have designed a parametric generator that uses a set of 7 morphological parameters. There are 7 major morphological parameters used for constructing geometries using the parametric generator: the maximum diameter $D_{\max}\in \left[35, 70\right]\;\mathrm{mm}$, the length $L_{a}\in \left[60, 120\right]\;\mathrm{mm}$, the eccentricity $e\in \left[0, 0.4\right]$, the wall thickness $t\in \left[1.2, 3.0\right]\;\mathrm{mm}$, the STJ ratio $r_{\mathrm{STJ}}\in \left[0.7, 1.0\right]$, the curvature asymmetry $\alpha_{c}\in \left[-0.3, 0.3\right]$, and the local bulge index $\beta \in \left[0, 0.5\right]$. These parameters are sampled using Latin hypercube design and each parameter configuration was meshed using the Gmsh library [51] with second-order tetrahedral elements resulting in 10,000 to 25,000 elements per configuration. We confirmed that the pairwise Pearson correlations of the 7 morphological parameters were all less than $\left|\rho \right|<0.06$, indicating that we sufficiently covered the design space with no unintentional clustering. The hyperelastic material characteristics of the geometries were determined from the published population distribution of Pasta et al. [52–54] and are defined by $\mu \in \left[15, 95\right]\;\mathrm{kPa}$, $k_{1}\in \left[0.5, 45\right]\;\mathrm{kPa}$, $k_{2}\in \left[0.1, 20\right]$, and $\kappa_{d}\in \left[0.226, 0.333\right]$. The reported errors for the test geometries derive from the same population distribution as that of the training set, rather than sampling its extremes, resulting in their representing average case performance.

We applied pressure loads to the geometries at a range of 80/60 mmHg (hypotensive) to 200/120 mmHg (severe hypertension) with an increment of 10 mmHg. The parametric generator produced a total of 1,247 geometries along with their associated results from FEA that were simulated using the open-source nonlinear FEA solver FEBio [55], which has been validated for soft-tissue mechanics, on an 8-core Intel Xeon Gold 6248 2.5 GHz central processing unit (CPU) for a duration of 12 to 45 min for each analysis. Additionally, further 86 FEA simulations from published ATAA research studies [11,13,17] were included. The publicly available solutions were standardized in format by calculating displacement and stress values at integration points, linearly interpolating onto common reference meshes, and verifying that they reproduced von Mises stress distributions that were within 3% relative error of published figures. The 3 source studies analyzed included mesh densities of 8,400 to 22,300 second-order tetrahedral elements using similar HGO-family constitutive models. A cross-validation of the 3 studies indicated that there was no significant bias between the studies above the threshold set for the standardization of results.

### MechToken encoding

The high density of raw stress field nodal values ($O(10^{4})$) makes it difficult for existing LLMs to process them without an excessive number of tokens. Here, we present the concept of “MechTokens”, or compact representations of stress fields that retain clinically pertinent characteristics while forming fixed-length vectors of the spatial distribution.

MechTokens are created through 3 processes: (a) Eight anatomical zones (anterior, posterior, left lateral, right lateral, greater curvature, lesser curvature, STJ, and mid-ascending) are created from the cylindrical coordinate system associated with the centerline of the ATAA surface, following established conventions for ascending aortic regional analysis [56]; we did not formally sweep over zone count, which we acknowledge as a limitation. (b) Five summary statistics of the von Mises stress are calculated for each zone (${\bar{\sigma }}_{z}$ [mean], $\sigma_{z}^{95}$ [95th percentile], $\sigma_{z}^{\max}$ [max], ${\mathrm{CV}}_{z}$ [coefficient of variation], and ${\left|\nabla \sigma \right|}_{z}$ [maximum magnitude of gradient]). (c) Two global features, the peak-stress-to-strength ratio (PSR) determined using the population mean strength reported by Martin et al. [13] and the ratio of peak stress predicted by Laplace’s law of a cylinder of equivalent diameter to peak stress. MechTokens have the form of the vector $m\in {\mathbb{R}}^{42}$ (8 zones · 5 statistics + 2 global features).

The elements of each MechToken are linearly projected to a 768-dimensional embedding and concatenated with the visual and text tokens of the LLM. Preliminary data from the experiment (shown in Table S12) demonstrated that zone-level summaries capture 94.1% of the variance in per-node stress distributions, whereas a single global mean captures only 61.3%.

### Multimodal LLM branch for conversational decision support

#### Backbone and instruction tuning

We extend the foundation laid by LLaVA-Med-v1.5 [57], a vision–language model composed of 7 billion parameters that has been trained using images and text within the realm of biomedical imaging. The initial phase in the construction of such a model consists of a visual encoder (CLIP ViT-L/14) that accepts as input the maximum-intensity projection (MIP) images corresponding to both the axial and sagittal views of the ATAA geometry. From the MIP renderings, the visual encoder generates 576 visual tokens. Upon generation of these visual tokens, they are combined with 42 MechToken embeddings, along with text tokens from the user’s specific query. These combined tokens are subsequently passed to the Vicuna-7B language decoder for final processing.

For the instruction-tuning process, 2 primary steps were taken. The first step was to freeze both the language and visual encoders and only train the MechToken projection layer and the cross-attention adapter (4 layers, 8 heads, 768 dimensions) on 2,160 single-turn question-answering (QA) pairs for a total duration of 5 epochs. Following this, the second tuning phase allowed all parameters to be fine-tuned using low-rank adaptation (rank 16, α=32) for a total of 3 epochs across the full 4,320-pair dataset. This dataset was constructed by combining information from 3 distinct sources: (a) statements from biomechanics textbooks and associated stress fields (1,440 pairs); (b) decision rules that had been derived from the 2010 American College of Cardiology Foundation/American Heart Association [3] and the 2014 European Society of Cardiology (ESC) recommendations [4] that had undergone adaptation to include reasoning based on stress; and (c) adversarial probes that had been developed specifically to investigate whether the model’s inference defaulted to diameter-only reasoning despite stress information contradicting diameter heuristics.

#### Conversational risk report generation

During inference, the LLM receives a structured prompt that contains the MechToken vector and ATAA geometry descriptors (e.g., diameter, length, and eccentricity), along with the simulated pressure and the user’s natural-language question. The system prompt provides specific instructions to the model: (a) summarizes both peak and regional stress distribution; (b) compares the PSR to population-based threshold values; (c) contextualizes the results in light of diameter-based guidelines; and (d) identifies and flags discrepancies in the stress- and diameter-based risk stratification. The model bases its output on the MechToken data and has been specifically trained to not create any false data when the model does not have access to the stress information.

### Training objective and joint optimization

The PINN branch and the LLM branch must be trained sequentially, rather than end to end, because it is necessary for the PINN to first reach convergence on its solution, which is consistent with physics, before producing output that is reliable enough to be used as conditioning for the LLM. The overall training process includes (a) training the PINN with the use of synthetic and publicly available FEA data; (b) generating stress predictions for the geometrical set of instruction tuning; (c) encoding the resulting stress predictions into an appropriate format (MechTokens); and (d) then tuning the LLM with these MechTokens.

Algorithm 1 summarizes the training procedure.


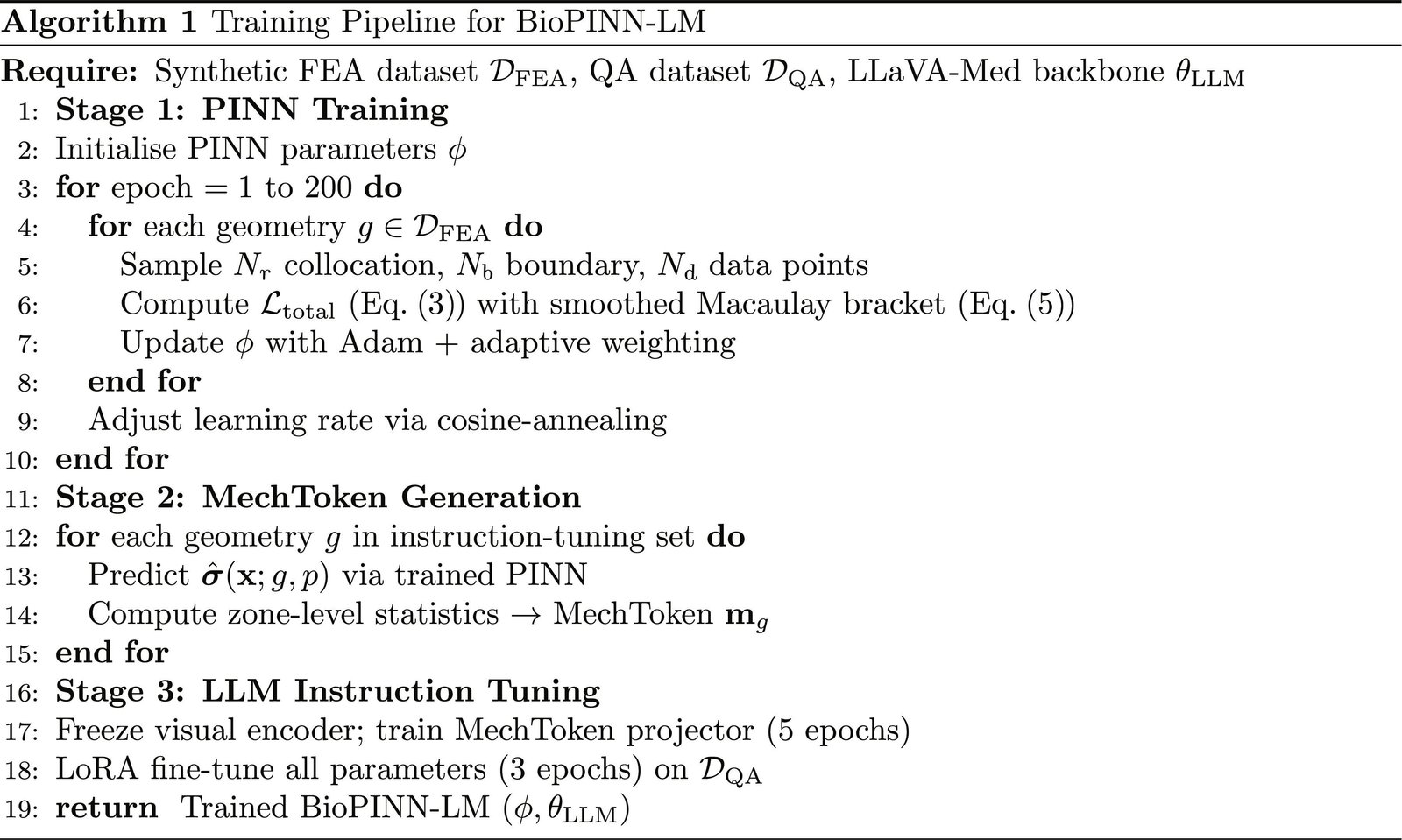


### Complexity analysis

The total number of floating-point operations (FLOPs) for a pinforward pass per geometry is equal to the number of collocation points multiplied by the amount of operations needed to evaluate the forward pass on the network per collocation point. In terms of depth and width of the network dimensions, this amount of operations is given as a function of *D* for depth and *W* for width equal to the number of collocation points evaluated on the network per pinforward pass. The number of collocation points is represented by *N*_r_ = 8,192. For the given parameters *D* = 8, *W* = 256, and *N*_r_ = 8,192, the approximate FLOPs incurred for the forward pass is 5.4 × 10^9^. The autograd operations incurred due to the need to calculate the quantities $\nabla \cdot \hat{P}$ via the constitutive law, as well as for the deformation gradient and HGO material response, will be about 3 times the effective cost of the FLOPs, providing for a measured wall-clock time of 0.83 s when using a single NVIDIA A100 graphics processing unit (GPU). The MechToken encoding due to its utility in the MechToken pipeline is somewhat negligible, incurring less than 5 ms. For each query, the 7-billion-parameter LLM receives a mean of 618 input tokens, corresponding to approximately 8.6 × 10^12^ FLOPs and 2.1 s of compute time on an A100 when quantized to 4 bits. The total pipeline latency per pinforward case is expected to be less than 3 s. In comparison, FEBio FEA for the same geometry takes an average of 38.6 min on 8 CPU cores.

## Results

### Datasets and experimental setup

**Fig. 4.**
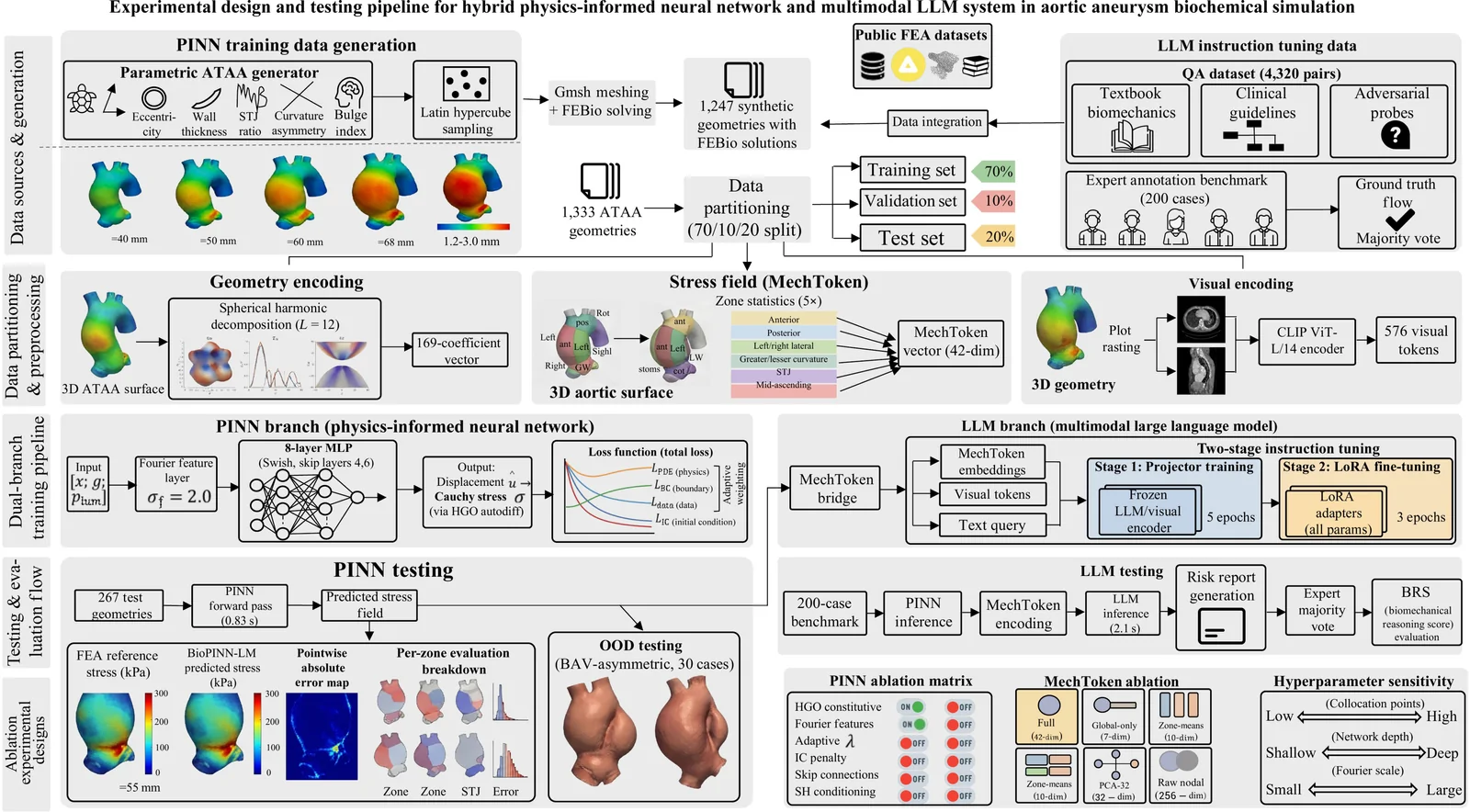
The experimental pipeline of BioPINN-LM. In the upper part of the figure is a geometric representation of synthetic ascending thoracic aortic aneurysm (ATAA) geometries, generated using a parametric sampling method and then solved using FEBio (left), as well as 4,320 question-answering (QA) pairs used for large language model (LLM) instruction tuning (right). The second part of the diagram shows how geometries can be encoded using spherical harmonics (left), summarized using MechToken’s stress field, and reproduced using CLIP’s image encoding methodology (right). The third section provides a detailed view of the architecture of the physics-informed neural network (PINN) branch and its loss through embedding the Holzapfel–Gasser–Ogden (HGO) model into the loss function, in addition to detailed instruction tuning of the LLM branch through a 2-stage process. The fourth section provides an overview of the testing and evaluation workflows for both branches of experimentation, including OOD morphological assessment using bicuspid aortic valves (BAVs). The final section provides information regarding the experimental designs of the ablation experiments (PINN component removal, MechToken encoding variants, and hyperparameter sensitivity sweeps).

Fig. 4 presents the complete experimental pipeline, illustrating the data generation, dual-branch training, evaluation, and ablation protocols adopted in this study.

#### PINN evaluation data

Of the total of 1,247 synthetic geometries plus 86 public solutions from the FEA, 70% (933), 10% (133), and 20% (267) of those became training, validation, and test datasets, respectively. Four groups were created according to the maximum diameter quartiles, which allowed each to maintain proportions of small, medium, large, and very large geometries. Despite the large number of geometries of 26 in the very large group ($D_{\max}>65\;\mathrm{mm}$), any conclusions about this subgroup should be construed accordingly (see Table S3). The test set contains 53 geometries (small aneurysms) with $D_{\max}<45\;\mathrm{mm}$ and 38 geometries (large aneurysms) with $D_{\max}>60\;\mathrm{mm}$, in order to assess generalizability/robustness across a range of clinical settings (Table S3). Additional out-of-distribution (OOD) testing was performed on 30 geometries that exhibited asymmetric dilation patterns associated with bicuspid aortic valves (BAVs) and were not used to train the model.

#### LLM evaluation data

We constructed a simulated clinical vignette benchmark using 200 simulated vignettes. To develop these simulated clinical vignette cases, we sampled ATAA geometry from our test set, then calculated stress predictions with the PINN model, and finally built simulated clinical vignette cases containing diameter, growth rate, comorbidities, and pressure profiles. The comorbid conditions used to construct the vignette cases (Marfan syndrome, hypertension, and BAV) were selected to reflect the published real-world distribution [4] of these diseases, rather than being purposely skewed toward pathological extremes. Approximately 40% of the simulated clinical vignette cases were within the 45- to 55-mm clinical gray zone as defined by Della Corte et al. [58], allowing us to evaluate the roles of ambiguous patterns in our model predictions. The clinical scenario vignettes were sent to 5 board-certified CV physicians. There were 3 cardiac specialists and 2 interventional cardiologists. The median years of practice after fellowship were 12. The physicians came from 2 different academic medical centers. The physicians independently gave management/clinical recommendations (monitoring, an increase in medical therapy, referral for surgery, and urgent intervention) for each of the cases according to a standardized annotation rubric developed specifically for this study. Each category of management/clinical recommendation as outlined in the rubric contained specific clinical criteria. Each clinician had to document the clinical rationale for their particular management/clinical recommendation. Each annotator was blind to the responses of all other annotators as well as the PINN model predictions. The majority vote for management demonstrates the ground truth in this study. There was moderate interrater agreement in this study (Fleiss’s *κ* = 0.71, 95% confidence interval [CI] [0.64, 0.78], indicating the reliability of the results from the 5 annotators). Pairwise Cohen’s κ for the 10 annotator pairs ranged from 0.62 to 0.78 with no single annotator identified as an outlier, indicating that moderate overall agreement was spread across the panel rather than due to specific rater disagreements.

#### Baselines

For stress prediction, we compared BioPINN-LM with reference FEA performed using FEBio, a vanilla PINN [19] without the physics of the hyperelastic HGO material model, a DeepONet [23], a PI-DeepONet [21], and a data-driven convolutional neural network surrogate based on a 3D U-Net architecture that has been trained on the same FEA data. For decision support based on LLMs, we used the following: GPT-4V with structured prompts (without any stress data), LLaVA-Med-v1.5 (without the MechToken feature), and an expert system encoded using a rule-based system based on the ESC 2014 guidelines.

#### Metrics

Prediction of stress using this model is summarized by the following metrics for nodal stress predictions: MAE (kPa), relative MAE (%), peak wall stress error (%), and coefficient of determination ($R^{2}$). The LLM was assessed using specialist consensus agreement (%), F1-weighted scoring for the 4 main recommendation types, and the biomechanical reasoning score (BRS). Two attending CV surgery specialists assigned the BRS on a 0-to-10 scale using 5 criteria (regional stress identification, peak stress-to-strength reasoning, guideline contextualization, discrepancy flagging, and presence of falsified values). Test–retest intrarater reliability was evaluated by an intraclass correlation coefficient (ICC) of 0.84, and interscorer reliability was evaluated by an ICC of 0.79 using a random subset of 40 cases.

#### Implementation details

Data processing for training both the PINN and LLM models occurred on a workstation running 4 NVIDIA A100 80-GB GPUs and 2 AMD EPYC 7763 processors and running PyTorch 2.1. The PINN’s HGO model was developed using custom back-propagation algorithms for calculating gradients with respect to the parameters of the model, including its smooth Macaulay brackets (Eq. (5)); the model could also learn through a combination of custom loss functions. The LLM was developed using the LLaVA codebase and was fine-tuned with DeepSpeed ZeRO-3 to reduce GPU memory requirements. Training comprised 200 PINN epochs over 47 h, followed by LLM stage 1 and stage 2 training over 6 and 14 h, respectively. For each of 3 independent runs, we used random seeds 42, 123, and 256 and report the average ± standard deviation (Table S5).

Table 1 summarizes the dataset characteristics.

**Table 1.** Dataset characteristics for BioPINN-LM evaluation

Dataset	Source	Size	Split	Purpose
Synthetic FEA	Parametric + FEBio	1,247	70/10/20	PINN training
Public FEA	[11,13,17]	86	Merged	PINN training
BAV-OOD	Parametric (asymmetric)	30	Test only	Robustness
QA pairs	Textbooks + guidelines	4,320	80/10/10	LLM tuning
Simulated vignette benchmark	Specialist-annotated	200	Test only	LLM evaluation

FEA, finite element analysis; PINN, physics-informed neural network; BAV, bicuspid aortic valve; OOD, out of distribution; QA, question-answering; LLM, large language model

### Main results: Stress prediction

**Fig. 5.**
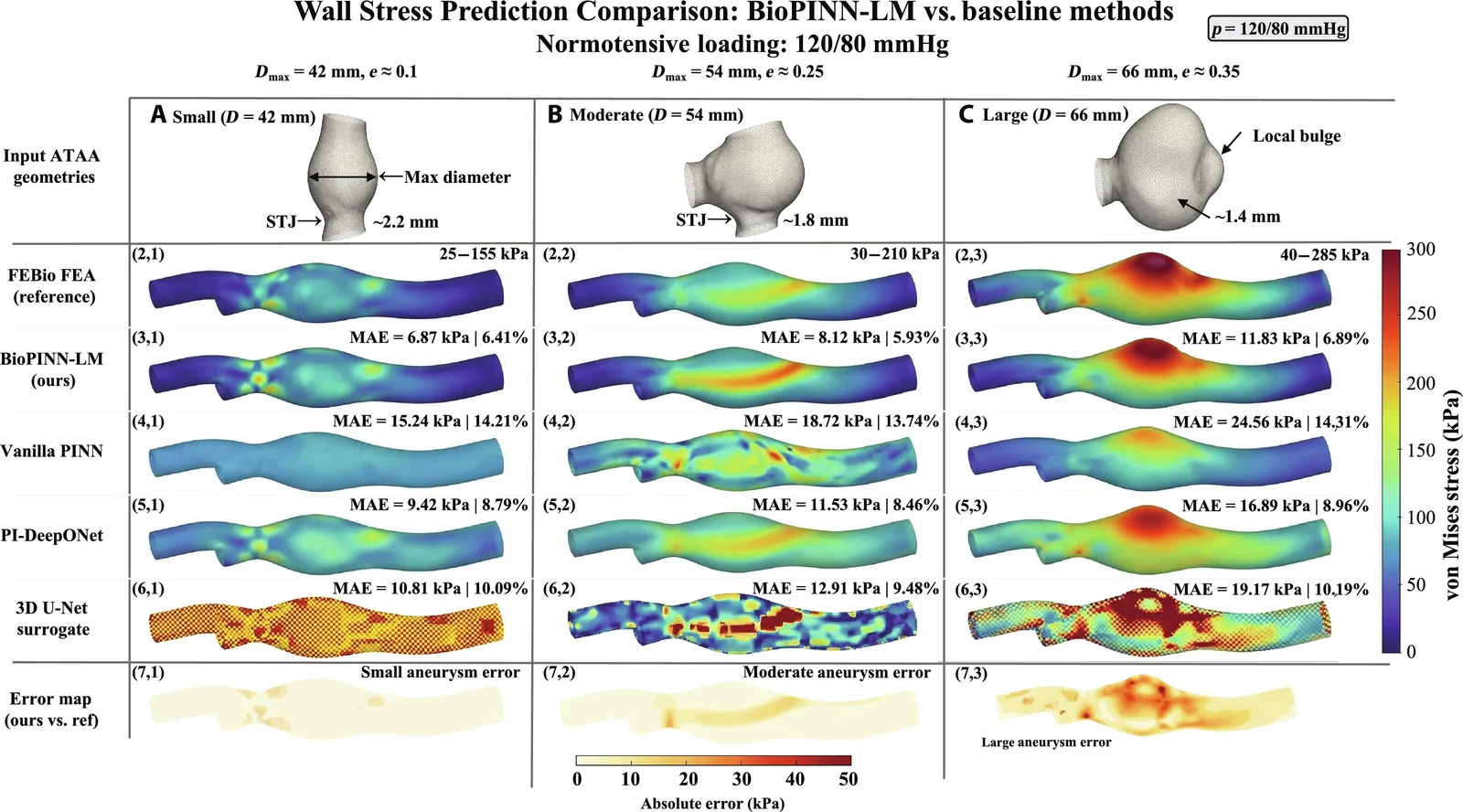
Evaluation of wall stress predictions for 3 different ascending thoracic aortic aneurysm (ATAA) geometries under normotensive loading (120/80 mmHg). For each column (A-C), the aneurysm size is specified: small ($D_{\max}=42\;\mathrm{mm}$), moderate ($D_{\max}=54\;\mathrm{mm}$), and large ($D_{\max}=66\;\mathrm{mm}$). The results are presented in the rows showing the following items arranged from top to bottom: input geometries, FEBio finite element analysis reference, BioPINN-LM prediction, vanilla physics-informed neural network (PINN) prediction, physics-informed deep operator network (PI-DeepONet) prediction, 3D U-Net surrogate prediction, and pointwise absolute error between our model and the reference. The inset table provides the average mean absolute error (MAE) and relative error calculated across all test geometries.

The accuracy of the PINN branch of BioPINN-LM compared to that of baseline methods for predicting stress under two pressures, normal blood pressure (120/80 mmHg) and high blood pressure (160/100 mmHg), is shown in Table 2. The qualitative differences between von Mises stress fields predicted for 3 typical ATAA geometries with increasing severity are shown in Fig. 5, along with error maps and a summary of each method’s accuracy.

**Table 2.** Wall stress prediction accuracy on 267 held-out ATAA geometries. Bold: best; underlined: second best. All values are mean ± standard deviation (SD) over 3 runs.

Method	120/80 mmHg (normotensive)				160/100 mmHg (hypertensive)			
	MAE/kPa	Rel./%	Peak/%	$R^{2}$	MAE/kPa	Rel./%	Peak/%	$R^{2}$
FEBio FEA (ref.)	—	—	—	—	—	—	—	—
Vanilla PINN ^a^	18.72 ± 1.3	13.74 ± 0.9	16.41 ± 1.8	0.871 ± 0.012	27.63 ± 2.1	14.68 ± 1.1	19.52 ± 2.3	0.849 ± 0.018
DeepONet ^a^	14.06 ± 0.9	10.32 ± 0.7	13.28 ± 1.4	0.912 ± 0.008	20.41 ± 1.6	10.85 ± 0.8	15.73 ± 1.7	0.893 ± 0.011
PI-DeepONet ^a^	11.53 ± 0.7	8.46 ± 0.5	10.17 ± 1.1	0.938 ± 0.006	16.89 ± 1.2	8.98 ± 0.7	12.84 ± 1.3	0.921 ± 0.009
3D U-Net surrogate	12.91 ± 1.1	9.48 ± 0.8	12.63 ± 1.6	0.923 ± 0.009	19.17 ± 1.8	10.19 ± 1.0	15.07 ± 2.0	0.902 ± 0.013
BioPINN-LM (ours)	**8.34 ± 0.6**	**6.12 ± 0.4**	**7.89 ± 0.9**	**0.961 ± 0.004**	**11.07 ± 0.8**	**5.89 ± 0.5**	**9.46 ± 1.0**	**0.953 ± 0.005**
W/o HGO	13.67 ± 0.9	10.03 ± 0.6	12.18 ± 1.2	0.927 ± 0.007	19.84 ± 1.4	10.55 ± 0.8	14.93 ± 1.5	0.908 ± 0.010
W/o adapt. λ	9.81 ± 0.7	7.20 ± 0.5	9.14 ± 1.0	0.951 ± 0.005	13.42 ± 1.0	7.13 ± 0.6	11.27 ± 1.2	0.940 ± 0.007

ATAA, ascending thoracic aortic aneurysm; MAE, mean absolute error; FEA, finite element analysis; PINN, physics-informed neural network; DeepONet, deep operator network; PI-DeepONet, physics-informed deep operator network; HGO, Holzapfel–Gasser–Ogden

^a^ Reproduced using author-released code.

An MAE of 8.34 kPa (6.12% relative), achieved by the full BioPINN-LM PINN at normotensive pressure, was 3.19 kPa (27.7% relative reduction) lower than PI-DeepONet’s MAE of 11.53 kPa and 4.57 kPa lower than 3D U-Net’s surrogate’s MAE of 12.91 kPa. Due to increasing stress magnitudes and increasingly pronounced constitutive nonlinearity as loading becomes hypertensive, BioPINN-LM’s overall improvement is even greater, 34.5% lower than the MAE of 16.89 kPa achieved by PI-DeepONet. The $R^{2}$ values of 0.961 (normotensive) and 0.953 (hypertensive) demonstrate high levels of linear agreement between BioPINN-LM’s predictions and FEA nodal data. A secondary analysis of peak wall stress using the Bland–Altman method shows a mean bias of −1.2 kPa with 95% limits of agreement of [−17.4,15.0] kPa at 120/80 mmHg, suggesting little to no pilot bias compared with FEA. These metrics establish BioPINN-LM as an accurate surrogate for the FEA pipeline, not a validated predictor of patient-level rupture or dissection risk: low PINN–FEA error supports computational fidelity, not clinical risk validity.

In contrast, the vanilla PINN, which enforces only linear-elastic equilibrium without the anisotropic HGO constitutive model, performed notably worse (MAE 18.72 kPa, 13.74%). This supports the statement that the ability to encode the constitutive law for specific problems is critical, not just a benefit.

### Main results: Conversational decision support

Table 3 reports the decision-support performance on the 200-case specialist-annotated benchmark. This benchmark tests whether the conversational interface yields specialist-plausible recommendations on simulated scenarios; it does not establish clinical validity, and results should be read as hypothesis generating rather than evidence of clinical translation.

**Table 3.** Conversational decision support performance on the 200-case simulated vignette benchmark. Concordance = agreement with specialist majority vote. BRS = biomechanical reasoning score (0-to-10 scale, blinded). Boldface identifies the best-performing value.

Method	Concordance/%	Weighted F1	BRS
ESC guideline rules	72.0	0.694	2.1 ± 0.4
GPT-4V (no stress)	74.5 ± 1.2	0.723 ± 0.018	3.7 ± 0.6
LLaVA-Med (no MechToken)	76.0 ± 1.5	0.741 ± 0.021	4.2 ± 0.7
LLaVA-Med + diameter only	78.5 ± 1.1	0.769 ± 0.016	4.8 ± 0.5
BioPINN-LM (ours)	**87.4 ± 0.9**	**0.862 ± 0.012**	**8.3 ± 0.4**
W/o MechTokens	77.3 ± 1.4	0.751 ± 0.019	4.5 ± 0.6
W/o adversarial QA	83.1 ± 1.0	0.819 ± 0.014	7.1 ± 0.5

ESC, European Society of Cardiology; QA, question-answering

BioPINN-LM has an 87.4% rate of agreement with specialists’ evaluations and is 15.4 points higher than the ESC rule-based system (72.0%) and 12.9 points greater than GPT-4V without stress data (74.5%). The BRS is at least 8.3, compared to 3.7 for GPT-4V, indicating that BioPINN-LM can provide biomechanical reasoning based on specific biomechanical locations or patterns, as well as present PSR, rather than focusing solely on the geometry or diameter of an object (Table S4).

The “W/o MechTokens” row represents a reduction in the ability of the BioPINN-LM model to perform biomechanical reasoning to 77.3% (similar to the performance of LLaVA-Med without any data related to stress), demonstrating that the genesis of performance related to biomechanical reasoning is due to the MechToken encodings as well as having trained on an adversarial question-answering methodology that will promote the nonbiomechanical or non-geometry-focused component of biomechanical performance to maintain the overall performance of BioPINN-LM being as high as possible. Notably, the diameter-only variant (78.5%) and the w/o-MechToken variant (77.3%) both collapse toward the diameter baseline, whereas the full model reaches 87.4%; together with the encoding ablations in Table S12 (global-stats-only 80.2% vs. zone-resolved 87.4%), this indicates the gain stems from regional biomechanical information rather than diameter or geometry alone. Within the gray-zone subset (45 to 55 mm, ∼80 vignettes), which dominates the discordance- and under-triage errors in Table S4, the regional MechToken signal contributes the largest relative gain over diameter-only baselines; we report this as a descriptive subgroup observation, and a formal gray-zone stratified analysis on a larger real CTA cohort is reserved for future work. The data in Table S4 show a wide CI due to category counts being very small (e.g., 2 in 25 hallucinated stress), so these are intended to be indicator numbers only rather than stable prevalence estimates. Seed standard deviation values indicate stochasticity due to initialization and collocation only and will not account for the sensitivity associated with the composition of the QA training set, which is another area of variability to explore independently.

### Ablation study: PINN components

Table S11 isolates the contribution of each architectural and training choice in the PINN branch.

The component of the HGO constitutive law embedding is most significant as shown by the increase in MAE by 5.33 kPa (63.9% decrease) if it were removed from the calibration (Table S1). The spherical harmonic geometry conditioning comes in as a close second component based on the amount the error increases when removed (+4.55 kPa) as this shows the need for representing the geometry in a compact, yet expressive way. Supervised displacement matching (SDM) provides an increase of +3.18 kPa and is consistent with the hybrid PINN paradigm in that it anchors the non-physics-based solution.

The presence of 2 distinct interaction effects indicates that each has differing relations/contributions to the overall error. The combined removal of both the HGO law and supervised data increases the MAE by 13.00 kPa, which is greater than just the sum of their individual contributions (5.33 + 3.18 = 8.51 kPa). Thus, it would appear that the HGO physical law and SDM provide complementary sources of constraints on the solution, making their simultaneous removal particularly debilitating. The combined removal of both Fourier and adaptive weighting produces an increase of +3.39 kPa, much less than the additive production of 1.94 + 1.47 = 3.41 kPa.

The individual contribution of skip connections was the least beneficial and fell short of the necessary level of statistical significance (*P* = 0.063; refer to Table S9). For the smaller aneurysm group ($D_{\max}<45\;\mathrm{mm}$), skip connections made no contribution to the overall MAE (ΔMAE<0.1kPa); however, for the larger aneurysm group ($D_{\max}>60\;\mathrm{mm}$), skip connections yielded a +1.34 kPa increase, probably due to the increased steepness of stress gradients at the STJ associated with larger geometric configurations.

### Ablation study: MechToken encoding

Table S12 compares MechToken variants to assess which statistics most impact LLM decision quality.

**Fig. 6.**
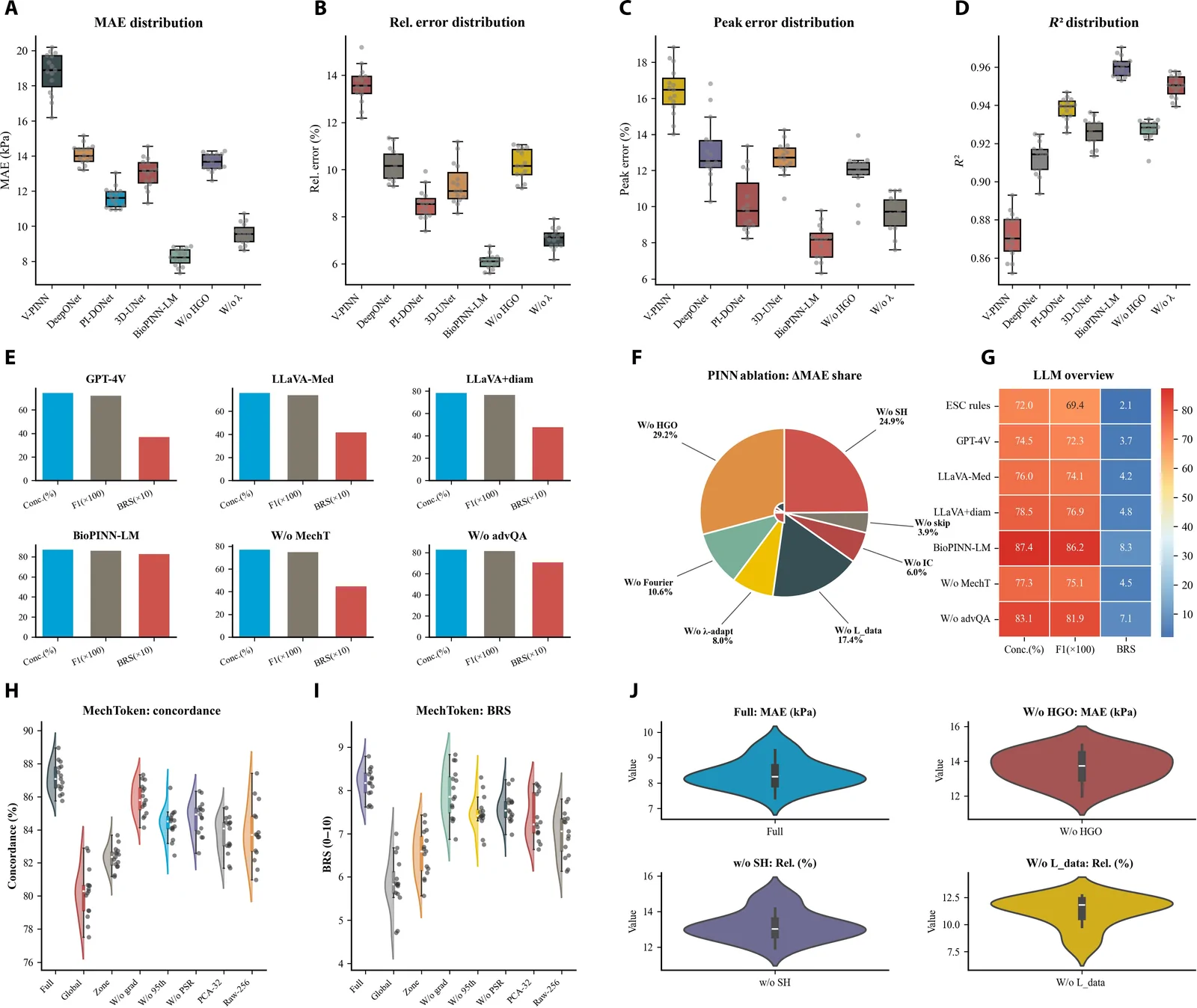
The below listings comprise Fig. 7, which provides an overview of the results obtained from the main experiments and ablation studies. Sections (A) to (D): Box plot distributions of mean absolute error (MAE), relative error, peak stress error, and *R*-squared values for all methods evaluated on 267 test geometries at 120/80 mmHg. Section (E): How the recommendation categories align with the specialist majority vote across 6 decision-support configurations on the 200-case benchmark. Section (F): Pie chart showcasing how all components contributed to MAE increase; each component is removed from a physics-informed neural network (PINN) configuration and its relative contribution to the total MAE increase. Sections (G), (H), and (I): Concordance, weighted F1, and biomechanical reasoning score across all large language model (LLM) variants and MechToken encoding variants. Section (J): The nodal MAE distributions for selected PINN ablation configurations.

The 42-dimensional feature-vector MechToken outperforms all compressed-model variants. Replacing zone-resolved descriptors with global statistics reduces accuracy by 7.2 percentage points, demonstrating the importance of spatial stress localization for LLM recommendations. The variant using 256 raw nodal samples performs worse than the structured 42-dimensional representation (84.1% vs. 87.4%), indicating that structured semantic content is more useful to the LLM than heterogeneous raw inputs. These observations are supported by earlier multimodal LLM studies showing that compact, interpretable feature vectors can benefit reasoning over raw signals [26]. Fig. 6 consolidates the PINN stress-prediction results (Fig. 6A to D, F, and J) and LLM decision-support results (Fig. 6E and G to I), providing a unified summary of the “Main results: Stress prediction” to “Ablation study: MechToken encoding” sections.

### Hyperparameter sensitivity

**Fig. 7.**
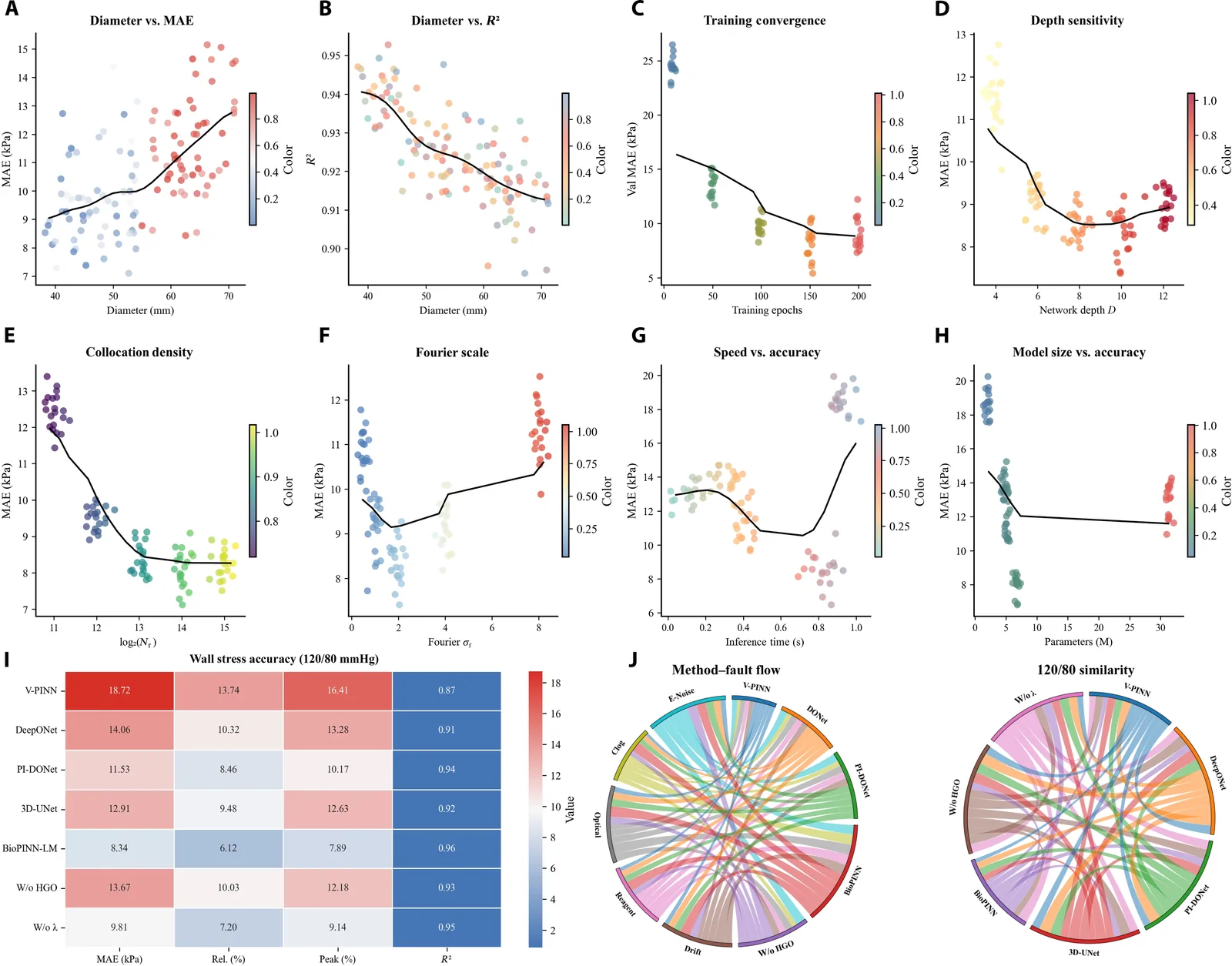
How well the physics-informed neural network (PINN)-based method has been evaluated in a comprehensive way regarding (a) geometric conditions, (b) behavior during training, (c) sensitivity to hyperparameters, and (d) computational trade-offs. In parts (A) and (B) of the figure, mean absolute error (MAE) and *R*-squared (*R*^2^) values are plotted against the maximum diameter (*D*_max_) of the aneurysm and the thinness of the wall, colored differently (red if thick and blue if very thin). Part (C) shows how the validation MAE decreased over time as the number of epochs (200) increased and illustrates this by drawing a line through the plotted MAE values. For parts (D) to (F) of the figure, the sensitivity of the MAE to the depth of the neural network (DNN), density of the collocation points, and scale of the Fourier features, respectively, have been assessed. Lastly, parts (G) and (H) compare the speed at which a PINN-based model can make predictions with the accuracy of those predictions as well as the size of PINN-based models relative to their accuracy. Part (I) provides a heatmap depicting the average stress accuracy for wall models created by various methods at 120/80 mmHg (normotensive loads), while part (J) depicts chord diagrams that show how stress patterns produced by these methods have significant variations and overlap with respect to their predictions during normotensive load conditions.

Table S6 examines the sensitivity of the PINN branch to key hyperparameters. There is a point of diminishing returns for a collocation density beyond $N_{r}=8,192$ because increasing the number of collocation points by 2 times to $N_{r}=16,384$ does not significantly improve the MAE (0.16 kPa); however, it takes nearly twice as long to complete the training. The results show that the depth of the network *D* has a nonmonotonic optimum at D=8, whereas deeper networks (D=10 and D=12) exhibit noticeable overfitting (as seen from the increased validation loss) even though they have slightly higher training accuracy than shallower networks (D=6 and D=8). The Fourier feature scale $\sigma_{f}$ has a very sharp optimum. An optimal value of $\sigma_{f}=2.0$ balances the competing goals of capturing the spatial resolution of stress gradients and minimizing the loss of accuracy due to spectral leakage. When using $\sigma_{f}=8.0$, the network has a tendency to try to fit oscillations that are very high frequency, near the boundary layers, which results in a decrease in accuracy to 11.36 kPa. Fig. 7 visualizes these hyperparameter sensitivities (Fig. 7D to F), diameter-stratified accuracy (Fig. 7A and B), training convergence (Fig. 7C), and computational trade-offs (Fig. 7G and H).

### Computational cost comparison

Table S7 compares computational costs across methods. The PINN branch by itself offers a speedup of 2,790 times compared to FEBio (0.83 s vs. 2,316 s); however, its training requires a longer duration of time (47 h). When all components of the complete pipeline including the LLM inference are combined, they produce results in less than 3 s, which is sufficient for interactive consultation. While the 3D U-Net surrogate can perform an inference faster than PINN (0.12 s), it does not have as much accuracy (MAE: 12.91 kPa vs. 8.34 kPa) and does not enforce physical laws, meaning that it is not going to be as effectively used when outside the range of what it has learned from the training set.

### Per-zone stress prediction breakdown

Table S8 reports per-zone MAE to identify where the PINN excels and struggles. The lowest error percentage for the PINN (4.83%) occurred in the highest curvature (i.e., the area of the arterial wall that has a relatively constant [and smooth] cylindrical shape) was expected, whereas, conversely, the highest error percentage (8.42%) occurred at the STJ. This high error is caused by the rapid changes in geometry between the ascending aorta and the aortic valve (complex stress concentration between them). The limits of PINN performance are demonstrated by this high error because the STJ region requires high-resolution FEAs that allow the local mesh refinement to resolve stress concentrations, whereas the combination of PINN’s meshless numerical solution and FEA does not allow for equivalent local refinement. In an additional study of different approaches to STJ error reduction, the introduction of an enrichment scheme for collocation points adjacent to the STJ reduced the STJ error to 7.16% at the expense of +0.15 s of added inference time (Table S2), which should be further studied.

### Statistical significance and robustness

Table S9 reports paired Wilcoxon signed-rank tests comparing BioPINN-LM against each baseline on the 267-geometry test set. Statistical significance is achieved in all comparisons made, excluding the comparison for the skip connection ablation. This independence from statistical significance is indicated by the marginal value of P=0.063, correlating with the low value of improvement found (+0.72 kPa) in Table S11. The comparison between the performance of the overall model and that of the PI-DeepONet results in a significant difference (P=0.003). The gap versus the vanilla PINN is larger still (P<0.001), whereas the narrower yet significant margin over PI-DeepONet confirms it as the most competitive baseline.

### Robustness analysis

Table S10 evaluates the PINN branch under distribution shifts: unseen pressure levels and BAV-associated OOD morphologies. When experiencing high blood pressure, performance decreases only slightly when using pressure extrapolation methods; at 200/120 mmHg, which is outside of the training range for the model, the relative error increased to 7.41%, but this result is still lower than vanilla PINN performance (13.74%). The relative error for BAV-OOD geometries is 9.83% ± 1.1% (bootstrap 95% CI, [7.81,11.85]%; *n* = 30), which shows that the transverse asymmetry was larger than that of the training distribution. As the BAV-OOD set is small (*n* = 30) and does not span CTA quality, segmentation, wall thickness, or solver/meshing variability, these robustness results should be regarded as preliminary rather than evidence of broad OOD generalizability. However, performing well below normal pressures does improve performance slightly compared with the training range; therefore, BAV geometries are not expected to outperform other types of BAV geometries. This behavior is consistent with the observation that less pressure results in less deformation of the model. Therefore, although all BAV-OOD geometries exhibit similar behavior at both lower and higher pressures, predictions were made near to and above the linear range where PINN approximations are most accurate.

## Discussion

The evidence shown in Results supports the idea that combining a PINN with a multimodal language model allows for the development of an efficient computational framework for the biomechanical prediction and support of clinical decision-making related to ATAA. Several different components of this research provide opportunities for further discussion.

### Biomechanical fidelity versus speed

At the level of normotensive loading, PINN’s mean relative error to FEBio FEA found through the use of subsecond inference (6.12%) is very similar in magnitude to interanalyst variability reported for manual FEA pipelines (5% to 10% [16]) and is considerably lower than the uncertainty from the images used to create the geometry (wall thickness estimation alone has been shown to have an uncertainty of 10% to 15% [59,60]). The approximation error from PINN generates not the most uncertainty for this pipeline. Because MechTokens and recommendations derive from the predicted stress field, upstream uncertainty propagates downstream: the dominant source is wall thickness/material uncertainty (10% to 15%) rather than the volumetric penalty (a 0.1- to 10-MPa sweep shifts peak stress <0.4%; Eq. (2)). Formal propagation to MechToken values and recommendation categories is left to future work; categories near the PSR threshold are the most uncertainty sensitive. However, it is noted here that the numerical references produced from the FEA technique do not relate to actual wall stress measurements generated from in vivo biomechanics; therefore, a match between PINN and FEA does not necessarily indicate actual risk of biological rupture or dissection. However, the increased error observed at the STJ (8.42%, Table S8) does represent a limitation for situations in which the STJ area contributes to the potential for rupture and therefore should be the subject of future study with respect to targeted collocation methods and adaptive refinement strategies [61].

### Value of constitutive law embedding

The removal of the HGO constitutive law (Table S11, +5.33 kPa) via ablation as well as the joint removal of HGO and data supervision (synergistic penalty: +13.00 kPa) demonstrates a more general phenomenon in biomechanical applications, specifically that domain physics are not simply a regularizer but also a structural prior. Therefore, the solutions produced by using domain physics must still be bounded to the possible range of solutions by domain physics. Without domain physics, networks must learn constitutive behavior purely from data. This is ultimately possible only if the training data are sufficient to represent all possible loading conditions as it is impractical for the field of aortic mechanics where the available testing data are quite limited and very costly to obtain. Fig. S1 provides Shapley-additive-explanations-based interpretability analysis confirming that the HGO material parameters (Fig. S1E to H) exert substantial influence on stress predictions, alongside the geometric and loading parameters (Fig. S1A to D), while Fig. S1I illustrates the comparative training dynamics across methods.

### From stress field to language

The MechToken encoding was created out of the necessity for current LLM architectures to work with fixed-length token sequences (therefore, the ingestion of tens thousands of nodal stress values is both impractical and ineffective). In the ablation shown in Table S12, it is shown that having 42 structured descriptors leads to greater reasoning than utilizing 256 raw nodal stress samples (i.e., 87.4% concordance versus 84.1% concordance). This suggests that for language models, structured semantics provide more opportunity for reasoning than raw data volume; these observations have also been noted within multimodal LLM literature, where task-related features created through engineering consistently outperform those obtained through direct injection of feature data [26].

### Limitations

A number of limitations need to be taken into account.

Firstly, the current use of a population-level constitutive parameter distribution, as used in the PINN formulation, rather than individual-specific parameters represents another source of error that would be reduced only through the availability of mechanical testing data on a patient-specific basis—which would be incompatible with the noninvasive aspiration of the framework. Future work could potentially address this limitation through the use of kinematic inversion methodologies to estimate the parameters for HGO from dynamic imaging [62].

Secondly, the 87.4% concordance was based on 200 simulated clinical scenario vignettes and not on actual longitudinally followed patients with ruptured or dissected aortas or surgical outcomes; therefore, the interrater agreement was moderate (0.71, 95% CI [0.64,0.78]), thus limiting both the power of any statistical analysis and its ability to be generalized to practice. Increasing the sample size to at least 500 cases, spread across a minimum of 3 institutions, would increase confidence in the concordance estimates.

Thirdly, the entire framework relies on static geometry derived from CTA data and does not include pulsatile loading data derived from the use of 4-dimensional (4D) flow MRI or cardiac-gated acquisitions; therefore, the PINN model would need to be modified to incorporate time-dependent (dynamic) loading for this purpose [22].

Fourthly, the smoothed Macaulay bracket (Eq. (5)) has been shown to have negligible approximation error for the range of stress magnitudes seen in this study and has not yet been validated at extreme loading regimes beyond 200/120 mmHg when the fiber engagement threshold is more significant.

Fifthly, several follow-up directions remain, ordered from internal model analysis to external validation: (a) stronger LLM controls (diameter-only, shuffled MechToken, and stress–diameter discordant subsets) to further confirm information beyond diameter, plus a formal characterization of the adaptive λ-weight trajectories; (b) a full biomechanical sensitivity analysis over wall thickness, loading, boundary conditions, and the zero-pressure assumption; (c) a dedicated 45- to 55-mm gray-zone stratified analysis [58]; (d) extension to newer medical LLMs [32]; and (e) a pilot study with ∼20 CTA-derived patients linked to longitudinal outcomes. Until these directions are addressed, BioPINN-LM should be considered a research prototype and not a viable clinical decision-support tool.

### Future directions

From a clinical standpoint, BioPINN-LM will serve as a supplementary biomechanical perspective for heart team multidisciplinary discussions, especially among those patients sitting in the 45- to 55-mm gray zone and those with Marfan/BAV risk modifiers [63]; additionally, once validated upon real CTA patient cohorts, this model could assist in determining when to surgically intervene, perform biomechanical risk stratification of patients beyond their aortic diameter, and identify at-risk patients who have moderate size aneurysms that still exhibit high wall stress responses. The above extensions would use 4D flow MRI data to permit PINNs to model the complete cardiac cycle and capture dynamic stress peaks during systole. This method may help PINN build a better prediction of when dissection occurs. The use of retrieval-augmented generation (RAG) with real-time access to clinical guidelines and database resources on the LLM side could decrease the risk of RAG hallucination and provide clinicians with the most current recommendations, without the need to retrain models for every update. The bionic systems community can build off these motivations to develop a broad effort to integrate mechanistic simulation with AI in other areas of research, such as soft robotic prostheses, implantable device development, and developing neuro-rehabilitation plans, where information from the mechanical modeling of systems is shared with multiple stakeholders.

## Conclusion

We have introduced a hybrid approach called BioPINN-LM that combines a PINN designed for the wall stress estimation of ATAA and a multimodal language model allowing physicians to converse with the system in an intuitive manner, as well as provide biomechanical insight into the clinical decision-making process.

Using HGO to establish the boundary conditions when calculating wall stress, our PINN prototype was able to complete model runs and provide wall stress estimates for 267 previously untested synthetic ATAA states in only 0.83 s per geometry, representing a speedup factor of 2,790. Consequently, the wall stress estimates had a mean error of 8.34 kPa (6.12% relative to the predicted value of normotensive loading) and had a mean error of 11.07 kPa (5.89% relative to hypertensive loading) at the time of analysis.

When evaluating the performance of the language model module, a group of specialists was used to evaluate our 200-case benchmark using the MechToken structure, and we found that 87.4% of their clinical recommendations were in accordance with the model output, which was 15.4 percentage points above the accuracy achieved by diameter-only rule-based systems. We further established through ablation studies that the mechanism of action behind our superior performance was largely due to having included an HGO constitutive law embedding in our PINN and encoding of the stress and loading conditions at the zone level in our MechToken system. All validation conducted as part of the present work is based on simulations; we have used a set of expert-annotated simulated clinical vignettes as the benchmark for our output, rather than a longitudinally validated cohort. Furthermore, before clinical use can occur, further validation against true CTA-derived cases with associated ruptures, dissections, and/or surgical endpoints will need to occur.

## Ethical Approval

The processes documented herein represent purely computational studies; no field-collected data and no human survey or bio-sample data were used; therefore, no institutional review board approval was required. The synthetic shape generator used to create geometric shapes was determined using population-wide statistics available in the literature [11,52]. The publicly available FEA outputs [11,13,17] were made available to researchers by the authors of these papers, have been extensively incorporated into the field of aortic biomechanics, and are widely used in the field. The expert assessment benchmark used to build the synthetic scenarios was constructed from fictitious examples created by the authors. Any subsequent utilization of this method for support in making clinical/deployment decisions of any kind will require clinical-specific validation and subsequent approval by regulatory authorities outside of the present computational study.

## Data Availability

The PINN branch’s source code, synthetic ATAA geometric generation tool, MechToken encoders, and LLM’s instructional fine-tuning codes can all be found at https://doi.org/10.48804/41RAQA. The repository contains pretrained model weights and QA datasets developed for LLM fine-tuning from textbooks and published standards. A list of the original public domain FEA datasets used in this research is referenced in Refs. [11,13,17].

## Supplementary Materials

Supplementary 1 Supplementary Text Tables S1 to S12 Algorithm 2 Fig. S1
