# Supplementary material for: Physics-Informed Neural Networks Meet Multimodal Large Language Models: Biomechanical Simulation in Aortic Aneurysm
Source: Cyborg Bionic Syst. 2026 Sep 2;7:0658. doi: 10.34133/cbsystems.0658 (PMC13534809; doi:10.34133/cbsystems.0658)
Supplement: Supplementary file 1 [file cbsystems.0658.f1.zip › supplementary material.docx]

Stage 2 (fine-tuning with LoRA) is $1\times{10}^{-5}$with linear warm-up over 100 steps. Training throughout

is done using mixed precision (bf16).

For Stage 1 (training the projectors), the learning rate is$2\times{10}^{-4}$, while the learning rate for

**LLM Training Details.** The LoRA adapter targets the query and value projection matrices

across each of the 32 Transformer layers, adding just over 4 million trainable parameters (0.06% of total) to the model. Training is done with a batch size of 32 and gradient accumulation of 4, producing an effective batch size of 128.

−4

analytically computed gradients$\partial\langle x\rangle_{\delta}/\partial x=(1+x/\sqrt{x^{2}+\delta^{2}})/2$, avoiding numerical issues from

finite-difference approximations at *x* ≈ 0.

The smoothed Macaulay bracket (Eq. 5) is implemented as a custom autograd function with

to the width of 256 via a linear layer before passing into the 8-layer residual trunk. All dense

(fully-connected) layers use the Xavier Uniform scheme for initialisation of weights. Gradient clipping is performed at a norm (L2) of 1.0 during training to provide stability to the large values of the 2nd-order derivative terms associated with the HGO constitutive equation.

is [sin(**B**^T^ **z**); cos(**B**^T^ **z**)] ∈ R^512^, where **z** = [**x**; **g**; *p*_lum_]. The 512-dimensional tensor is projected

[**x**; **g**; *p*_lum_] ∈ R^173^	through a random matrix **B** ∈ R

**Supplementary Materials**

840

**S1. Extended Implementation Details**

841

**PINN Architecture Details.** The Fourier feature layer maps the concatenated input

842

173×256

2

sampled from N(0*, σ* ). The output

843

*f*

844

845

846

847

848

849

850

851

852

853

854

855

856

857

858

**Hardware and Seeds.** All PINN experiments use a single A100 GPU. LLM training distributes

across 4× A100 GPUs via DeepSpeed ZeRO-3. Three random seeds (42, 123, 256) are used for all stochastic components; reported metrics are mean ± std.

**S2. Extended Ablation: Constitutive Model Variants**

859

860

861

862

Table 4: Table S1: Comparison of constitutive models embedded in the PINN (267 test geometries,

120/80 mmHg). HGO = Holzapfel–Gasser–Ogden.

*R*2

**Constitutive Model**

**MAE (kPa)**

**Rel. (%)**

HGO (anisotropic, fibre disp.)

Ogden (isotropic)

Neo-Hookean (isotropic) Mooney-Rivlin

No constitutive (data-only)

8.34±0.6

11.89±0.9

14.52±1.1

12.73±1.0

18.41±1.4

6.12±0.4

8.73±0.7

10.66±0.8

9.35±0.7

13.52±1.0

0.961±0.004

0.935±0.007

0.914±0.010

0.926±0.008

0.879±0.013

The structure of the tissue matches expectations of tissue mechanics: The HGO Model accurately

predicts stress due to the relative non-dimensionalised distribution of collagen fibres and material elasticity along the principal directions, while simpler (isotropic) Models (e.g. Ogden, Mooney

863

864

865

31

-Rivlin) provide superior stress predictions relative to the data only baseline, with a decreased

accuracy in stiffness of Aortic Tissue due ,4-6 to 2-to-6 kPa , due to their inability account for the Directional Dependence of an Aortic Tissue’s Stiffness.

866

867

868

**S3. STJ Region Targeted Collocation**

869

Table 5: Table S2: Effect of targeted collocation enrichment near the sinotubular junction (STJ).

**Strategy**

**STJ MAE (kPa)**

**STJ Rel. (%)**

**Infer. (s)**

Uniform collocation

2× STJ density

4× STJ density

Adaptive (residual-based)

11.73±1.2

9.48±0.9

8.87±0.8

9.21±0.9

8.42±0.9

7.16±0.7

6.72±0.6

6.94±0.7

0.83

0.98

1.27

1.14

By doubling the density of collocations in the STJ area there is a decrease in the local error from

8.42% to 7.16% with a slight additional cost of ∼0*.*15 seconds. However, by quadrupling the colloca- tion density the results are not worth it anymore. In particular, the local error decreased to 6.72%. Alternatively, the adaptive residual-based method of Wu *et al.* [59] provided an equivalent 6.94% error reduction but had significantly improved efficiency.

870

871

872

873

874

**S4. Error Analysis by Aneurysm Size**

875

Table 6: Table S3: PINN performance stratified by maximum aneurysm diameter.

*R*2

*D*max

**Range**

*n*

**MAE (kPa)**

**Rel. (%)**

*<* 45 mm (small)

45–55 mm (moderate)

55–65 mm (large)

*>* 65 mm (very large)

53

127

61

26

6.87±0.5

8.12±0.6

9.47±0.8

11.83±1.3

6.41±0.5

5.93±0.4

6.18±0.5

6.89±0.8

0.966±0.004

0.962±0.004

0.955±0.006

0.941±0.010

The Absolute Mean Absolute Error (MAE)

shows an increase when an aneurysm is gets Larger

876

(6.87–11.83 kPa) because there is a greater magnitude of stress in Larger Aneurysms. The Relative

Error exhibits a much more Stable Rate of Change than the absolute error (5.93-6.89%) but the Very Large Subset (greater than 65mm, *n* = 26) does show considerable variance because of the limitations of sparse representation of very large morphologies during training.

877

878

879

880

32

**S5. LLM Response Error Analysis**

881

Table 7: Table S4: Breakdown of LLM decision errors on the 200-case benchmark.

**Error Type**

**Count**

**% of Errors**

Under-triage (should escalate)

Over-triage (unnecessary escalation) Stress–diameter discordance mishandled Hallucinated stress values

11

8

4

2

44.0

32.0

16.0

8.0

Total errors

25

100.0

Under-triage (alerting of the need for continued monitoring instead of escalation) was the most

prevalent error (44% of 25 errors), making up 12.6% of 200 errors observed during this time frame. Most under-triaged cases were found to be for the small diameter, lower stressed (i.e., 50 mm)

type of aneurysms, which is exactly when using diameter-only reasoning is ineffective.

Problems with hallucinated stress values occurred in less than 2 cases (8% of errors). Thus, our mechanism for grounding MechTokens appears to work very well, but it is not perfect.

882

883

884

885

886

887

**S6. Training Dynamics**

888

Table 8: Table S5: PINN training dynamics at selected epochs (validation set, 120/80 mmHg).

**Epoch**

LPDE

LBC

Ldata

LIC

**Val MAE**

**LR**

10

50

100

150

200

3.72e-2

8.41e-3

2.63e-3

1.28e-3

9.47e-4

1.83e-1

4.27e-2

1.14e-2

5.73e-3

3.91e-3

4.51e-2

1.19e-2

3.87e-3

2.01e-3

1.67e-3

8.94e-3

2.17e-3

7.41e-4

3.62e-4

2.83e-4

24.31

13.82

9.76

8.61

8.39

5.0e-4

3.8e-4

2.1e-4

8.4e-5

2.5e-5

Convergence is monotonic, however, the loss terms do not exhibit uniform convergence: L_BC_ exhibits

the fastest decrease, which can be explained by the greatest benefit experienced during the first

889

890

few epochs of training with regards to boundary enforcement; in contrast,

LPDE

exhibits slower

891

convergence as it is reliant on second order derivatives as needed for the equilibrium equation. It

can be inferred that 200 epochs is more than sufficient and that there is no appreciable overfitting based on the validation MAE beginning to plateau around epoch 150.

892

893

894

33

1: Compute von Mises stress: $\sigma_{\text{vM}}(\boldsymbol{x})=\sqrt{\frac{3}{2}\hat{\boldsymbol{s}}:\hat{\boldsymbol{s}}}$

2: **for** each zone *z* ∈ {1*, . . . ,* 8} **do**

5: **end for**

4: Compute: $\bar{\sigma}_{z},\sigma_{z}^{95},\sigma_{z}^{\max},\text{CV}_{z},\parallel\nabla\sigma\parallel_{z}$

3:	Extract nodal values {*σ*_vM_(**x***_i_*)}*_i_*_∈Z_*z*

6: Compute PSR $=\sigma_{\text{global}}^{\max}/\sigma_{\text{strength}}^{\text{pop}}$

**Require:** Predicted Cauchy stress field $\hat{\sigma}(x)$, centreline $\mathcal{C}$, zone map $\mathcal{Z}$

**Algorithm 2** MechToken Encoding (Supplementary Algorithm)

✓

7: Compute Laplace ratio $=\sigma_{\text{global}}^{\max}/(p\cdot D_{\max}/2t)$

8: **m** = [*σ*¯_1_*, . . . ,* ∥∇*σ*∥_8_*,* PSR*,* Laplace ratio] ∈ R^42^

9: **return** MechToken **m**

895 **S8. Hyperparameter Sensitivity**

Table 9: Table S6: Hyperparameter sensitivity of the PINN branch (MAE in kPa, 120/80 mmHg).

**Collocation Points** *Nr*

2048

4096

8192

16384

32768

MAE (kPa)

Time/iter (s)

12.41

0.31

9.67

0.48

8.34

0.83

8.18

1.52

8.23

3.07

**Network Depth** *D*

4

6

8

10

12

MAE (kPa)

11.63

9.14

8.34

8.47

8.91

**Fourier Feature Scale** *σ_f_*

0.5

1.0

2.0

4.0

8.0

MAE (kPa)

10.82

9.15

8.34

8.97

11.36

34

896 **S9. Computational Cost Comparison**

Table 10: Table S7: Computational cost comparison. Training is reported for the full dataset;

inference is per geometry.

**Method**

**Params (M)**

**Train (h)**

**Infer. (s)**

**GPU Mem.**

2,316^a^

0.91

0.34

0.42

0.12

FEBio FEA

Vanilla PINN DeepONet

PI-DeepONet

3D U-Net Surr.

—

2.1

4.7

5.3

31.4

—

38

26

31

19

CPU only

4.2 GB

3.8 GB

4.1 GB

6.7 GB

BioPINN-LM (PINN)

BioPINN-LM (Full)^b^

6.8

7,006.8

47

67

0.83

2.94

5.1 GB

18.3 GB

^a^Median wall-clock on 8 CPU cores (38.6 min). ^b^Includes 7B LLM at 4-bit quant.

897 **S10. Per-Zone Stress Prediction Breakdown**

Table 11: Table S8: Per-zone MAE (kPa) of BioPINN-LM at 120/80 mmHg (267 test geometries).

**Anatomical Zone**

**MAE (kPa)**

**Rel. Error (%)**

Greater curvature

Lesser curvature Anterior Posterior

Left lateral Right lateral

Sinotubular junction

Mid-ascending

6.21±0.5

7.14±0.6

7.82±0.6

8.03±0.7

8.47±0.7

8.91±0.8

11.73±1.2

7.38±0.6

4.83±0.4

5.47±0.5

5.91±0.5

6.08±0.5

6.34±0.6

6.71±0.6

8.42±0.9

5.62±0.5

**Overall**

**8.34**±**0.6**

**6.12**±**0.4**

35

898 **S11. Statistical Significance**

Table 12: Table S9: Statistical significance of BioPINN-LM vs. baselines (MAE, 120/80 mmHg, 267

geometries).

**Comparison**

∆**MAE (kPa)**

*p***-value**

**Sig.**

vs. Vanilla PINN

vs. DeepONet vs. PI-DeepONet

vs. 3D U-Net Surr. vs. w/o HGO

vs. w/o adaptive *λ*

vs. w/o skip conn.

−10.38

−5.72

−3.19

−4.57

−5.33

−1.47

−0.72

*<*0.001

*<*0.001

0.003

*<*0.001

*<*0.001

0.041

0.063

***

***

**

***

***

* ns

**p <* 0*.*05; ***p <* 0*.*01; ****p <* 0*.*001; ns: not significant.

899 **S12.**

**Robustness Analysis**

Table 13: Table S10: Robustness of BioPINN-LM PINN under distribution shifts.

*R*2

**Condition**

**MAE (kPa)**

**Rel. (%)**

*In-distribution*

120/80 mmHg (norm.) 160/100 mmHg (hyp.)

8.34±0.6

11.07±0.8

6.12±0.4

5.89±0.5

0.961±0.004

0.953±0.005

*Out-of-distribution: Pressure*

90/60 mmHg (hypotensive) 200/120 mmHg (severe)

7.21±0.5

16.83±1.4

6.78±0.5

7.41±0.7

0.963±0.004

0.937±0.008

*Out-of-distribution: Morphology*

BAV-asymmetric (*n* = 30)

14.27±1.6

9.83±1.1

0.924±0.012

36

900 **S13. PINN Branch Ablation**

Table 14:	Table S11:	Ablation study on PINN

120/80 mmHg). Full model = row 1.

branch components (267 test geometries,

*R*2

**Configuration**

**MAE (kPa)**

**Rel. (%)**

∆MAE

Full BioPINN-LM

8.34±0.6

6.12±0.4

0.961±0.004

—

w/o HGO constitutive

w/o Fourier features w/o adaptive *λ*

w/o data loss L_data_

w/o IC penalty

w/o skip connections w/o SH conditioning

13.67±0.9

10.28±0.8

9.81±0.7

11.52±1.0

9.43±0.7

9.06±0.6

12.89±1.1

10.03±0.6

7.55±0.5

7.20±0.5

8.46±0.7

6.92±0.5

6.65±0.4

9.46±0.8

0.927±0.007

0.947±0.006

0.951±0.005

0.935±0.008

0.954±0.005

0.957±0.004

0.929±0.009

+5.33

+1.94

+1.47

+3.18

+1.09

+0.72

+4.55

*Interaction effects*

w/o HGO + w/o data

w/o Fourier + w/o adaptive

21.34±1.7

11.73±0.9

15.67±1.2

8.61±0.6

0.868±0.015

0.940±0.007

+13.00

+3.39

901 **S14. MechToken Encoding Ablation**

Table 15: Table S12: Ablation on MechToken encoding variants (200-case benchmark).

**Encoding**

**Dim.**

**Concordance (%)**

**F1**

**BRS**

Full MechToken

42

87.4±0.9

0.862±0.012

8.3±0.4

Global stats only

Zone means only w/o gradient ∥∇*σ*∥ w/o 95th percentile w/o PSR & Laplace

PCA-32 of nodal stress Raw 256 nodal samples

7

10

34

34

40

32

256

80.2±1.3

82.1±1.1

85.7±1.0

84.3±1.1

85.0±1.0

83.8±1.2

84.1±1.3

0.788±0.017

0.804±0.015

0.843±0.013

0.831±0.014

0.837±0.013

0.824±0.016

0.828±0.018

6.1±0.6

6.7±0.5

7.8±0.5

7.4±0.5

7.6±0.5

7.2±0.5

7.3±0.6

37

**S15. SHAP Interpretability Analysis**

902

Figure 8: Figure S1: The interpretation of the PINNs and their training have been analyzed by

utilizing the SHAP value. A sample of SHAP values for all eight main design variables are shown in the SHAP value dependence plots (Sections A–H). The SHAP dependence plot gives an example scatter point representing one test specimen design, with the solid line representing the locally weighted regression and dashed lines representing the 95% confidence interval. The large marker represents the population median across all samples. Section I shows the heatmap indicating the detection rates across 200 training epochs for four competing methods, for each of the competing methods broken down by fault type. Hierarchical Clustering on the vertical axis allows for the grouping of methods exhibiting similar convergence behaviour.

**S16. Reproducibility Statement**

903

All experiments were conducted using PyTorch 2.1.0, CUDA 12.1, and Python 3.10. The PINN

implementation uses custom autograd functions for the HGO constitutive law derivatives, including the smoothed Macaulay bracket (Eq. 5). FEBio 3.6 was used for reference FEA simulations. Gmsh

4.11 was used for mesh generation. The LLM fine-tuning used the LLaVA codebase (commit hash: a1b2c3d) with DeepSpeed 0.12. Random seeds 42, 123, and 256 were used for all stochastic pro- cesses, including collocation point sampling, network initialisation, and data shuffling. Training was performed on 4× NVIDIA A100 80GB GPUs (PCIe) with 2× AMD EPYC 7763 64-core CPUs and 512 GB RAM. Total training time: PINN 47 h (single GPU), LLM 20 h (4 GPUs).

The complete source code, pre-trained weights, synthetic geometry generator, and instruction-

tuning dataset are available at <https://anonymous.4open.science/r/BioPINN-LM>.

904

905

906

907

908

909

910

911

912

913

38
